# Experience-dependent shaping of hippocampal CA1 intracellular activity in novel and familiar environments

**DOI:** 10.7554/eLife.23040

**Published:** 2017-07-25

**Authors:** Jeremy D Cohen, Mark Bolstad, Albert K Lee

**Affiliations:** 1 Janelia Research Campus, Howard Hughes Medical Institute, Ashburn, United States; Boston University, United States

**Keywords:** hippocampus, CA1, place cell, spatial memory, intracellular, whole-cell, Mouse

## Abstract

The hippocampus is critical for producing stable representations of familiar spaces. How these representations arise is poorly understood, largely because changes to hippocampal inputs have not been measured during spatial learning. Here, using intracellular recording, we monitored inputs and plasticity-inducing complex spikes (CSs) in CA1 neurons while mice explored novel and familiar virtual environments. Inputs driving place field spiking increased in amplitude – often suddenly – during novel environment exploration. However, these increases were not sustained in familiar environments. Rather, the spatial tuning of inputs became increasingly similar across repeated traversals of the environment with experience – both within fields and throughout the whole environment. In novel environments, CSs were not necessary for place field formation. Our findings support a model in which initial inhomogeneities in inputs are amplified to produce robust place field activity, then plasticity refines this representation into one with less strongly modulated, but more stable, inputs for long-term storage.

**DOI:** http://dx.doi.org/10.7554/eLife.23040.001

## Introduction

The hippocampus is a major component of the mammalian brain’s machinery for learning and memory (Andersen et al., 2007). In humans, hippocampal damage results in an inability to convert the ongoing experience of items and events from daily life into long-term memories (Scoville and Milner, 1957). In rodents, the hippocampus has been shown to be critically involved in encoding spatial layouts and locations of importance (O'Keefe and Conway, 1978; Morris et al., 1982). Neural representations of novel (i.e., previously unencountered) environments form rapidly in the rodent hippocampus (Hill, 1978; Wilson and McNaughton, 1993; Frank et al., 2004; Leutgeb et al., 2004) and consist of place cells with location-selective ‘place field’ spiking (O'Keefe and Dostrovsky, 1971). Place field activity stabilizes (i.e., becomes more similar across successive traversals of the environment) with experience (Wilson and McNaughton, 1993; Frank et al., 2004; Leutgeb et al., 2004; Cacucci et al., 2007), and the persistence of fields over time provides the neural basis of spatial memory (O'Keefe and Conway, 1978; Thompson and Best, 1990; Liu et al., 2012; Mankin et al., 2012; Ziv et al., 2013).

What processes underlie the formation of hippocampal memory representations? Synaptic plasticity has been strongly implicated in spatial learning-based behaviors (Morris, 1989; Tsien et al., 1996) as well as changes in place field activity (Bostock et al., 1991; McHugh et al., 1996; Mehta et al., 1997, 2000, 2002; Ekstrom et al., 2001; Lever et al., 2002; Cacucci et al., 2007; Bittner et al., 2015). However, the role of plasticity in creating new place cell representations is unresolved.

First, is plasticity necessary for place cell activity in novel environments? Pharmacological and genetic manipulations of NMDA receptors (NMDARs) suggest that synaptic plasticity is not needed for place fields to appear in novel environments (McHugh et al., 1996; Kentros et al., 1998). However, intracellular recordings suggest that specific, plasticity-inducing events are required to generate each new place field (Bittner et al., 2015). In particular, intracellular experiments have shown that large, calcium-mediated complex-spike (CS) events (Kandel and Spencer, 1961; Wong and Prince, 1978; Traub and Llinás, 1979; Harvey et al., 2009; Epsztein et al., 2011; Grienberger et al., 2014), which trigger synaptic plasticity in vitro (Takahashi and Magee, 2009) and are sufficient to artificially create place fields, always co-occur with the spontaneous appearance of new fields in familiar environments (Bittner et al., 2015). Since, by definition, all place fields in novel environments are new, does their appearance require CSs?

Second, does plasticity strengthen place fields with experience? Within a behavioral session, hippocampal CA1 place field firing rates increase with repeated traversals through each location (Mehta et al., 1997, 2000), which is consistent with a strengthening of the inputs to fields via Hebbian (Hebb, 1949) and spike timing-dependent (Markram et al., 1997; Bi and Poo, 1998) plasticity mechanisms. Across days, though, the opposite occurs, with the average firing rate of CA1 place cells being lower in familiar compared to novel environments (Nitz and McNaughton, 2004; Karlsson and Frank, 2008). In each case, what changes are occurring to the underlying inputs?

Third, what underlies the stable representation of familiar spaces? The low and sometimes variable (Mankin et al., 2012; Ziv et al., 2013; Rubin et al., 2015) firing rates of CA1 place fields in familiar environments work against having a stable representation of space. However, when a place cell fires, the reliability of where it fires increases with experience (Wilson and McNaughton, 1993; Leutgeb et al., 2004; Cacucci et al., 2007). What changes in inputs produce this overall stability?

Addressing these questions requires measuring the inputs to individual place cells and CSs while animals form new spatial representations, and after environments have become familiar. Therefore, we performed whole-cell intracellular recordings of hippocampal neurons in mice exploring both novel and familiar environments – a comparison which has previously been limited to extracellular recording. Unlike extracellular recording, intracellular recording allows access to inputs via the subthreshold membrane potential (V_m_), to CSs, and to intrinsic cellular properties such as the AP threshold that shape how inputs are converted into spiking output. Our experiments employed virtual reality-based methods in which animals moved through visually defined virtual environments by running in place (Hölscher et al., 2005; Harvey et al., 2009; Chen et al., 2013; Ravassard et al., 2013), which facilitated switching between different environments during intracellular recording. We assessed experience-dependent changes in subthreshold inputs and other intracellular features within a single exploration session in a given environment. We compared findings from sessions in novel environments to those in environments that had become familiar through multiple exploration sessions across several days. The results provide answers to specific plasticity-related questions and, more generally, help to bridge cellular and systems approaches to hippocampal learning. Overall, the findings support a model in which novel environments induce a rapid amplification of input activity in CA1, then the spatial tuning of inputs becomes less strongly modulated, but more reliable, across the entire environment with experience. The result provides the basis for a stable hippocampal representation of familiar spaces.

## Results

### A model of hippocampal spatial memory formation

**Figure 1.**
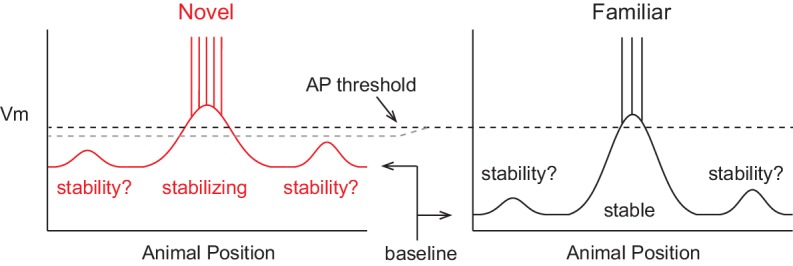
A model of intracellular changes underlying the formation of hippocampal CA1 place cell representations. Hypothesized intracellular features underlying place field activity in novel and familiar environments that are consistent with previous studies. Each side represents the membrane potential (V_m_) as a function of the animal’s location in a 1-dimensional environment. With repeated traversals of the environment, inputs underlying place field spiking are strengthened due to Hebbian or spike timing-dependent plasticity, which is reflected in a larger amplitude depolarization of the V_m_ from the baseline V_m_ level in familiar environments. The experimentally observed higher AP rate during novel experience is explained by a more depolarized baseline, which could result from the known reduction in CA1 inhibitory interneuron firing rates in novel environments. A lower AP threshold could potentially also contribute to higher AP rates in novel environments (lower, gray dotted line). The spatial tuning of inputs within the place field region is presumed to stabilize with experience, possibly due to plasticity triggered by intracellular complex spikes (CSs). Previous experimental data does not clearly inform what may occur regarding the stability of inputs outside of the field. Figure 7 shows our results from testing the features of this model with intracellular recordings. **DOI:** http://dx.doi.org/10.7554/eLife.23040.002

A model of the formation of place cell representations that fits previous experimental observations is shown in Figure 1. It shows the firing rate and subthreshold V_m_ of a CA1 place cell in both novel and familiar environments as a function of the animal’s location. Here, the spatially tuned inputs driving place field spiking are modeled as being initially small, then, due to plasticity, growing with experience (McHugh et al., 1996; Mehta et al., 1997, 2000; Savelli and Knierim, 2010; D'Albis et al., 2015). The observed decrease in firing rate when the environment has become familiar is hypothesized to be due to a lower baseline V_m_, resulting in reduced spiking output in spite of a larger input-based subthreshold V_m_ hill in the place field. The lower baseline could arise from the observed increase in inhibitory interneuron firing rates with familiarization (Wilson and McNaughton, 1993; Frank et al., 2004; Nitz and McNaughton, 2004). The inputs driving initial place field firing would be present from the onset of exploration and be based on the existing synaptic weight matrix without the need for CSs or plasticity, consistent with the finding that the majority of place fields are present during the first traversal of a novel environment (Hill, 1978). Instead, CS occurrence during some of the traversals (laps) across the environment could serve to strengthen inputs for long-term place field stability. This is in agreement with experiments showing that NMDAR-dependent plasticity is not required for place field formation during novel environment exploration, but is necessary for place field stability across days (Kentros et al., 1998). The larger subthreshold hill relative to out-of-field inputs would provide the basis for a reliable and long-lasting representation of familiar environments. We tested the features of this model with intracellular recordings of place field activity in behaving animals.

### Subthreshold V_m_ hill amplitude grows during novel environment exploration

**Figure 2.**
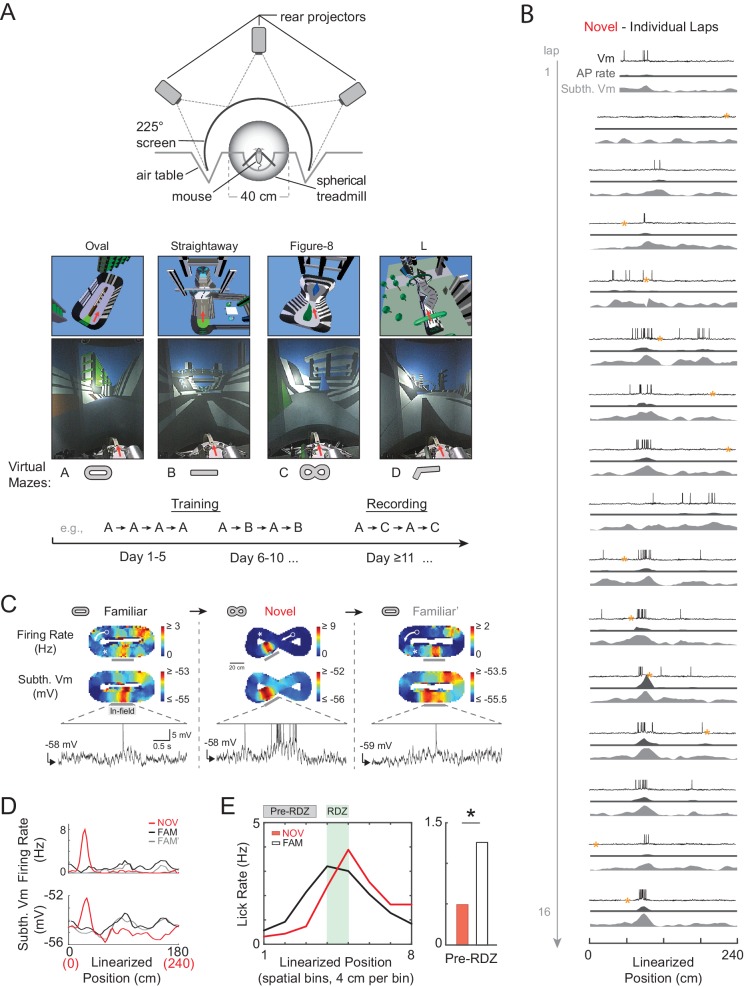
Whole-cell intracellular recording of mouse hippocampal CA1 neurons in novel and familiar virtual maze environments. (**A**) Top: Top view of virtual reality apparatus showing mouse on a spherical treadmill surrounded by an image of a maze environment projected onto a cylindrical screen (225° arc). Middle: The four virtual maze environments used in the study. Overhead view of 3-D scene models of the mazes (above). Photos of the rear-projected virtual mazes on the cylindrical screen taken from above and behind the animal position (below). Arrow shows location and perspective of the animal in the virtual maze (above) and on the spherical treadmill (below). Bottom: Example behavioral training and recording protocol. (**B**) Example whole-cell intracellular recording in a novel maze. V_m_ (top), AP rate (middle), and subthreshold V_m_ (bottom) for the first 16 laps in linearized position coordinates. Yellow asterisks mark times (every 20 s) when current injection was applied to probe series and input resistance and evoked spiking response. Note: ‘Subthreshold V_m_’ refers to the V_m_ after APs and CSs have been removed. (**C**) Activity of same cell shown in (**B**) as the animal first explored a familiar maze (left), then a novel maze (middle), then was re-exposed to the initial familiar maze (right). Overall AP rate (top) and subthreshold V_m_ (middle) in each of these three epochs. Note: ‘Epoch’ refers to the entire continuous period in a given maze. White arrow: Running direction. White asterisk: Primary reward location. Example V_m_ traces (bottom) from single traversals (gray bars) through place fields in each epoch (APs truncated). (**D**) Overall epoch activity from (**C**) in linearized coordinates. (**E**) Left: Lick rate in spatial bins immediately surrounding primary reward zone (RDZ) in novel and familiar mazes. Right: Predictive licking behavior in spatial bins immediately preceding RDZ (Pre-RDZ lick rate in Hz) in familiar versus novel mazes. *p<0.05. **DOI:** http://dx.doi.org/10.7554/eLife.23040.003

Whole-cell current-clamp recordings of dorsal hippocampal CA1 pyramidal neurons were obtained in adult head-fixed mice as they navigated around novel (NOV) and familiar (FAM) 1-dimensional (1-D) virtual maze environments linked to a spherical treadmill (Figure 2A–B; 32 cells from 15 mice; mean ± SD, recording duration: 8.3 ± 7.1 min, recording duration/epoch: 4.3 ± 4.0 min, laps/epoch: 8.9 ± 6.0; maze lengths: 135–240 cm; ‘epoch’ refers to the entire continuous period in a given maze). Virtual reality (VR) facilitated switching between distinct environments during intracellular recording (Figure 2C–D), while the head-fixed behavior in the track-like mazes yielded repeated exposures to all spatial locations within an environment without backtracking. Each cell’s location-based activity with respect to a common reference (the fixed-location primary reward zone) was uncorrelated across distinct mazes (Figure 2—figure supplement 1), suggestive of global remapping (O'Keefe and Conway, 1978; Muller and Kubie, 1987; Leutgeb et al., 2005) between the virtual environments. The animals’ licking shifted from occurring just after reward delivery at the primary reward zone in novel environments to occurring just before delivery (i.e., predictively) in familiar environments (Figure 2E and Figure 2—figure supplement 2A), providing behavioral evidence of spatial learning.

**Figure 2—figure supplement 1.**
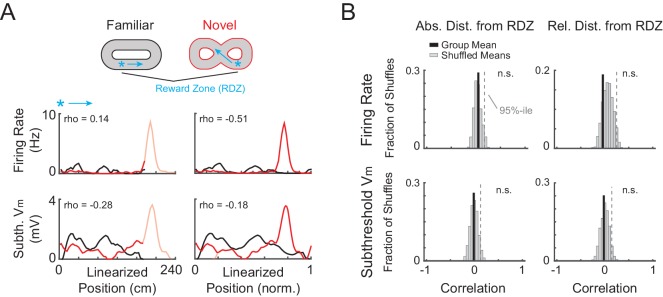
AP rate and subthreshold V_m_ activity aligned to the primary reward zone is not correlated between novel and familiar mazes. (**A**) Example activity from the NOV-FAM maze pair shown in Figure 2C, but here the linearized activity is aligned to the primary reward zone (RDZ, asterisk) in each maze. The linear correlation (Pearson’s r) for each maze pair comparison (left: absolute position, right: relative position) is noted. Arrow: Running direction. (**B**) For all cells exposed to NOV-FAM maze pairs (and active in both epochs for the firing rate correlation, and active or silent for the subthreshold V_m_ correlation), the mean of the spatial correlation scores (Group Mean, solid black) for the overall epoch AP rate and subthreshold V_m_ profile across pairs of mazes is plotted in absolute (left) and relative (right) coordinates aligned to the primary reward location. Group mean scores were compared to distributions of shuffled mean values (Shuffled Means, n = 5000). Dashed line marks the 95%-ile value in the shuffled distributions, showing that the spatial profile of the firing rate or subthreshold V_m_ was not correlated with respect to absolute or relative distance from the primary reward location. The lack of correlation suggests that a rate remapping-like process did not occur between the familiar and novel virtual mazes, and therefore that the activity in the novel virtual mazes was new. **DOI:** http://dx.doi.org/10.7554/eLife.23040.004

**Figure 2—figure supplement 2.**
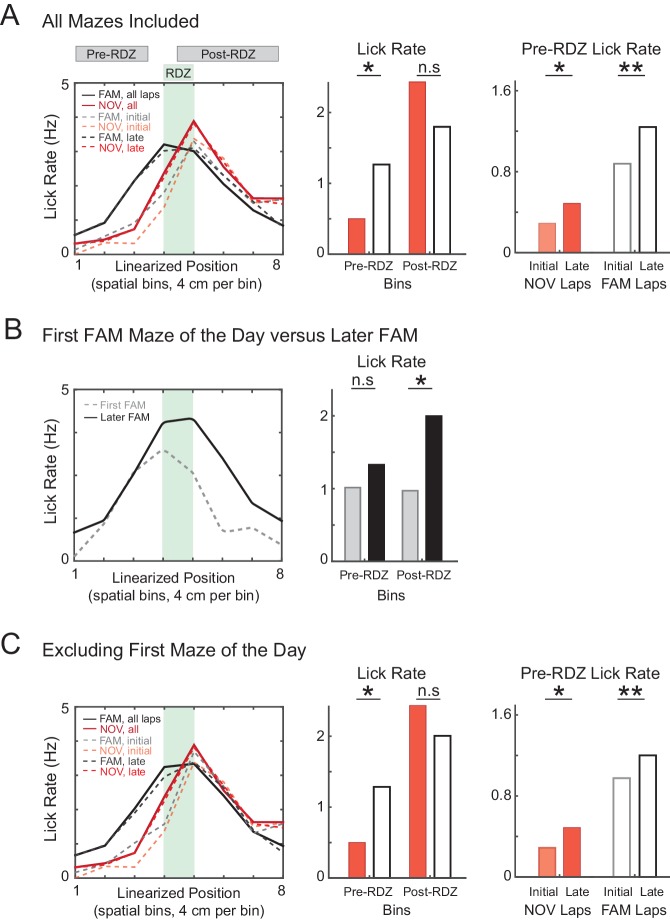
Increased predictive licking behavior with experience. (**A**) Lick rate in the spatial bins immediately surrounding the primary reward zone (RDZ). The amount of predictive licking is greater in familiar compared to novel mazes, and also increases during novel and familiar epochs, indicating that animals learned the location of the reward zone. (**B**) Same as (**A**) for the subset of FAM mazes that were also the first maze experienced by the animal that day, and separately for all other FAM mazes. The amount of predictive licking was similar for both subsets of mazes, but the amount of licking in response to receiving the reward was reduced in first mazes of the day, suggesting a difference in behavioral engagement. (**C**) Same as (**A**) excluding all mazes that were also the first maze experienced by the animal that day. *p<0.05; **p<0.01. **DOI:** http://dx.doi.org/10.7554/eLife.23040.005

**Figure 3.**
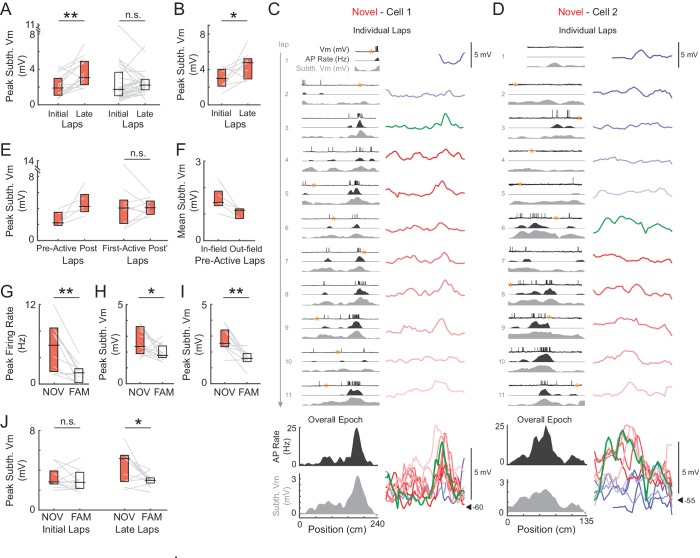
Subthreshold V_m_ depolarizations underlying hippocampal CA1 place fields grow in novel environments and are larger in novel compared to familiar mazes. (**A**) Peak subthreshold V_m_ in the initial laps 1–2 versus the late laps 6-end for all novel (NOV, left) and familiar (FAM, right) mazes. The peak refers to the peak value within the field determined from the epoch-averaged subthreshold V_m_ as a function of location. Here and elsewhere: Box marks 25-75th percentile values, horizontal line marks median. (**B**) Peak subthreshold V_m_ within the (spiking) place field in the initial versus late laps for all place fields in novel mazes. (**C**)-(**D**) Examples of place fields that formed in a lap later than the first lap in a novel maze. Subthreshold V_m_ for each lap at expanded (and fixed) voltage scale (right) and overlaid (bottom right). (**E**) Left: Peak subthreshold V_m_ within the place field in the laps before (Pre-Active) versus after (Post) the first lap with spiking in the field. This includes only those place fields in novel mazes that formed in a lap later than the first lap. Note that Post includes the first lap with spiking in the field. Right: Peak subthreshold V_m_ within the place field in the first lap with spiking in the field (First-Active) versus in the laps afterwards (Post’). This includes all place fields in novel mazes, including those that first spiked within the field in lap 1. In (C, right) and (D, right): Pre-Active laps (shades of blue), First-Active lap (green), Post’ laps (shades of red), Post laps (green and shades of red). (**F**) Mean subthreshold V_m_ inside versus outside the field in the laps before place field spiking for the novel maze place fields in (E, Left). (**G**) Overall epoch peak firing rate for all recorded pairs of novel and familiar mazes (NOV-FAM maze pairs) in which cell was active in at least one of the mazes in the pair. (**H**) Overall epoch peak subthreshold V_m_ for all NOV-FAM maze pairs whether or not cell was active or silent in either maze. (**I**) Same as (**H**) except for NOV-FAM maze pairs in which the cell had a place field in at least one of the mazes in the pair. (**J**) Peak subthreshold V_m_ within the V_m_-defined field in the initial laps 1–2 (left) and late laps 6-end (right) of all NOV-FAM maze pairs. P-values for paired comparisons from Wilcoxon signed-rank tests. *p<0.05; **p<0.01. **DOI:** http://dx.doi.org/10.7554/eLife.23040.006

We first checked the amplitude of the main subthreshold V_m_ hill during individual maze epochs for evidence of increases in synaptic strength. In novel environments, the peak amplitude increased between the initial (1–2) and late (6-end) laps (Figure 3A, left, 3B), similar to the number of laps over which substantial changes in place fields have been observed in extracellular studies (Mehta et al., 2000; Ekstrom et al., 2001). This was the case when considering the peak subthreshold V_m_ within the field determined from the average subthreshold V_m_ as a function of location over the entire epoch (median ± SE, NOV initial laps: 1.9 ± 0.5 mV, late laps: 3.1 ± 0.6, p=0.003, n = 14, Figure 3A, left), or, to allow for spatial jitter, the peak in each lap irrespective of location (‘lap peak’, NOV initial laps: 3.0 ± 0.4 mV, late laps: 4.0 ± 0.6, p=0.004). The increase in amplitude is consistent with a strengthening of inputs to place fields via Hebbian, spike timing-dependent, or other (Golding et al., 2002; Dudman et al., 2007) mechanisms of synaptic plasticity, and indicates rapid learning in CA1 in novel environments. In contrast, no increases in amplitude were observed within familiar mazes (p=0.47, Figure 3A, right; lap peak: p=0.70).

**Figure 3—figure supplement 1.**
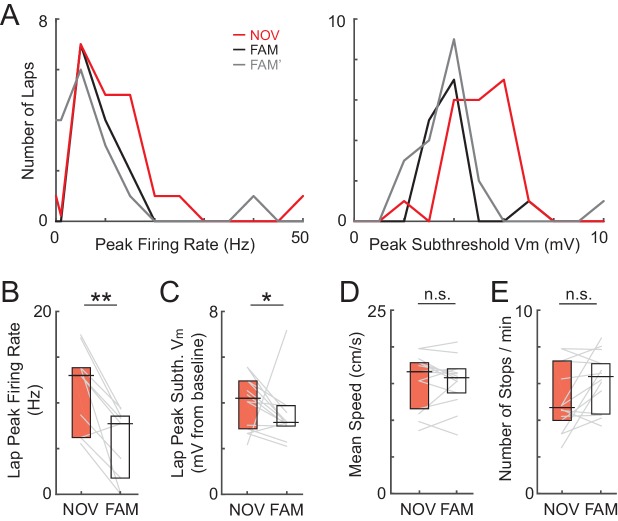
Higher AP rate and larger amplitude subthreshold V_m_ responses during novel exploration are not due to differences in running speed or stopping behavior. (**A**) Peak AP firing rate and subthreshold V_m_ amplitude during each lap in the epochs shown in Figure 2B–C. Note the right-shifted distribution of the peaks in the novel (NOV) compared to the two familiar maze epochs (FAM/FAM’). (**B**) Mean of the peak AP rate values irrespective of position during individual laps in each maze epoch for NOV-FAM maze pairs. (**C**) Same as (**B**) for peak subthreshold V_m_. (**D**) Overall epoch mean speed (cm/s) in the maze for all cells recorded during exposure to a NOV-FAM maze pair. (**E**) Same as (**D**) for overall epoch mean rate of stopping behavior (speed <2 cm/s for >1 s; number of stops/min). *p<0.05, **p<0.01. **DOI:** http://dx.doi.org/10.7554/eLife.23040.007

**Figure 3—figure supplement 2.**
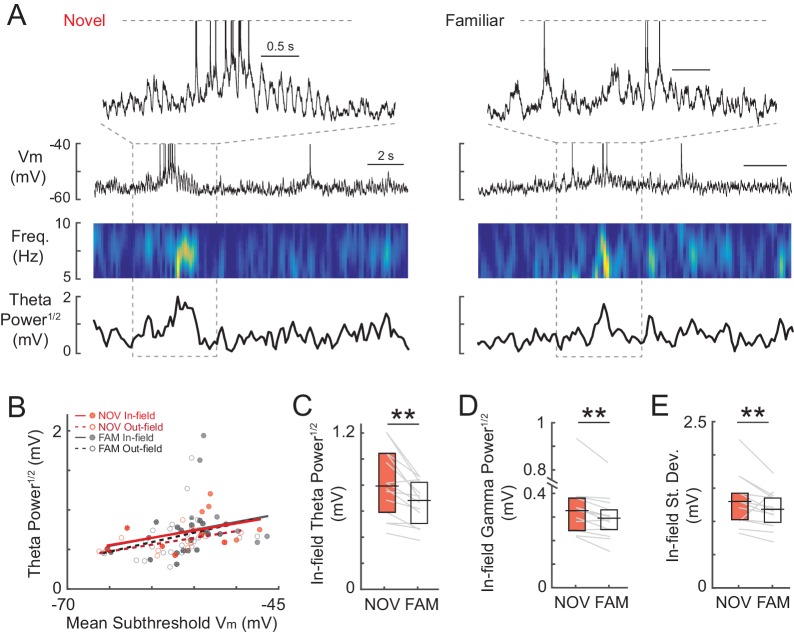
Intracellular membrane potential oscillations are larger during novel maze exploration. (**A**) Example V_m_ activity from a single cell during one lap in a NOV (left) and FAM (right) maze of a maze pair. Top traces show a close-up of the V_m_ activity inside the place field. Lower panels from top to bottom show the V_m_ trace, theta-band spectrogram of the subthreshold V_m_, and theta power^1/2^ of subthreshold V_m_ for the entire lap, respectively. (**B**) For all cells, theta power^1/2^ versus the mean subthreshold V_m_ inside (filled circles, solid lines) and outside (open circles, dashed lines) the field within NOV and FAM mazes. (**C**) Mean in-field theta power^1/2^ of subthreshold V_m_ for all cells recorded in a NOV-FAM maze pair. (**D**)-(**E**) Same as (**C**) for gamma power^1/2^ and standard deviation of subthreshold V_m_. **p<0.01. **DOI:** http://dx.doi.org/10.7554/eLife.23040.008

To further address possible mechanisms, we examined the subthreshold V_m_ amplitude with respect to the first lap with spiking in the place field. We decomposed the novel maze increase in peak subthreshold V_m_ amplitude within the place field region (NOV initial laps: 3.0 ± 0.7 mV, late laps: 4.8 ± 0.8, n = 9, p=0.039, Figure 3B; in contrast, FAM initial laps: 3.9 ± 1.3, late laps: 3.0 ± 0.6, n = 10, p=0.63) into two parts. For the subset of novel epochs in which place field spiking did not occur in the first lap, the peak subthreshold V_m_ in the place field increased sharply from the laps before to the laps after spiking began (Figure 3C–D and E, left). However, there was no consistent increase after the first spiking lap, including cases in which place field spiking occurred in the first lap of the epoch (First-Active lap: 4.0 ± 0.9 mV, Post’ laps: 4.1 ± 0.5, n = 9, p=1.0, Figure 3E, right). The sharp increase suggests mechanisms of plasticity that do not rely on postsynaptic spiking (Golding et al., 2002; Dudman et al., 2007). The lack of increase in peak subthreshold V_m_ amplitude after the first spiking lap was also observed for place fields in familiar mazes (p=0.42). In all five cases in which place field spiking was not present in the first lap of a novel epoch, the mean V_m_ in the laps before spiking was greater inside versus outside the eventual place field (Figure 3F; also see Figure 3C–D, bottom right). This suggests that inputs were biased towards the locations of the place fields before spiking occurred.

### Subthreshold V_m_ hill amplitude is smaller in familiar environments

We then compared the amplitude of subthreshold V_m_ hills in familiar versus novel environments. First, in agreement with previous extracellular recordings from freely moving rats (Nitz and McNaughton, 2004; Karlsson and Frank, 2008), the peak firing rate of active cells was higher in novel environments (peak of average firing rate as a function of location over the entire epoch, NOV: 5.9 ± 2.1 Hz, FAM: 1.7 ± 0.5, n = 10 NOV-FAM maze pairs with at least one epoch being active, p=0.004, Figure 3G; lap peak: NOV: 13.0 ± 2.3 Hz, FAM: 7.7 ± 2.1, p=0.004, Figure 3—figure supplement 1B), and the proportion of cells that were active was greater (69% of NOV epochs and 47% of FAM epochs).

**Figure 4.**
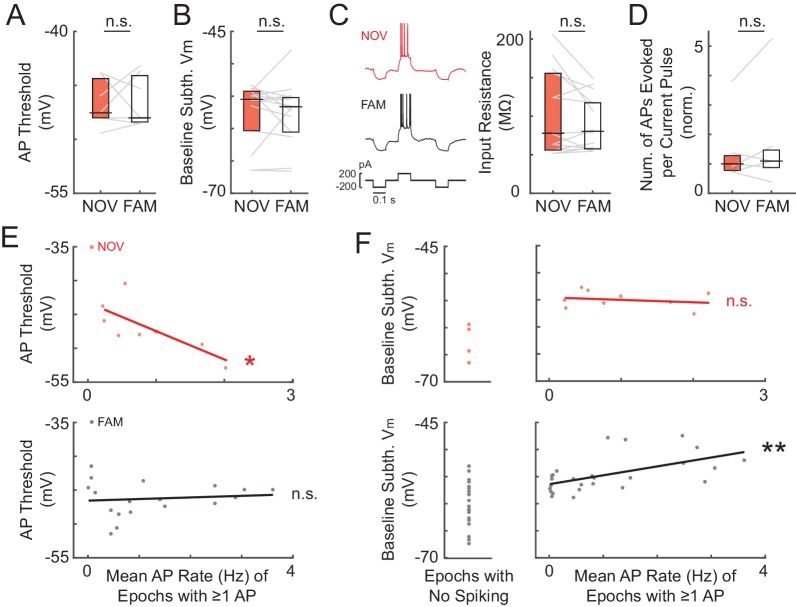
Somatic excitability does not differ between novel and familiar mazes. (**A**) Mean AP threshold for all cells recorded in a NOV-FAM maze pair that were active in both mazes. (**B**) Overall epoch baseline subthreshold V_m_ for all cells recorded in a NOV-FAM maze pair. (**C**) Left: Example intracellular current injection sequence and responses (to probe R_N_ and evoked firing) from a NOV-FAM maze pair. Right: Mean somatic R_N_ for all cells recorded in a NOV-FAM maze pair. (**D**) Mean number of APs evoked by the positive step in the current injection sequence (normalized to median value for each step size). (**E**) Mean AP threshold (mV) versus AP rate (Hz) for all NOV (top) and FAM (bottom) maze epochs with ≥1 AP. (**F**) Left: Baseline subthreshold V_m_ (mV) for all NOV (top) and FAM (bottom) epochs with no spontaneous APs. Right: Baseline subthreshold V_m_ versus mean AP rate for all NOV (top) and FAM (bottom) epochs with ≥1 AP. The baseline V_m_ was more depolarized in epochs with ≥1 AP than completely silent epochs (NOV: p=0.002, FAM: p<0.001). *p<0.05; **p<0.01. **DOI:** http://dx.doi.org/10.7554/eLife.23040.009

According to the model in Figure 1, the strength of place field inputs should either continue to increase or remain strengthened with additional experience, with the lower firing rates in familiar environments being explained by a more hyperpolarized baseline V_m_ and/or higher action potential (AP) threshold. However, we found that the spike threshold (Figure 4A) and baseline (Figure 4B) did not differ between novel and familiar environments. Instead, the amplitude of the peak subthreshold V_m_ was smaller in familiar environments (NOV: 2.4 ± 0.4 mV, FAM: 1.8 ± 0.2, n = 14 active and silent NOV-FAM maze pairs, p=0.042, Figure 3H; lap peak: p=0.002, Figure 3—figure supplement 1C; active maze pairs subset: NOV: 3.2 ± 0.4 mV, FAM: 1.9 ± 0.2, n = 8, p=0.023; note that including only active pairs controls for the lower proportion of active cells in familiar mazes; peak in place field: NOV: 2.5 ± 0.4 mV, FAM: 1.6 ± 0.1, n = 10 maze pairs with at least one epoch having a place field, p=0.004, Figure 3I). Furthermore, there was no sign that any part of the increase in amplitude during novel exploration remained by the time environments became familiar (p=0.52, Figure 3J, left). The second highest subthreshold V_m_ peak in each environment was not, however, larger in novel compared to familiar environments (NOV: 1.4 ± 0.1 mV, FAM: 1.4 ± 0.1, n = 14, p=1.0).

**Figure 4—figure supplement 1.**
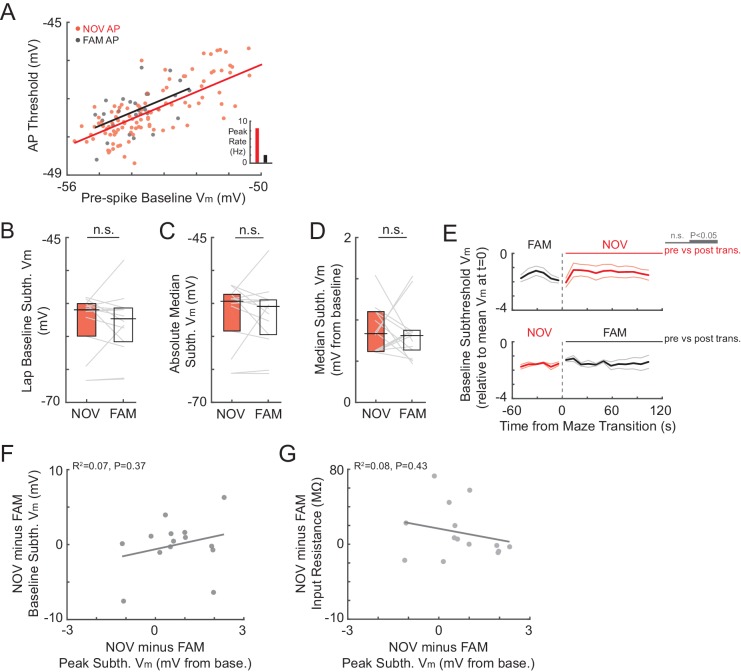
No differences in AP thresholds, somatic baseline V_m_, or input resistance can account for the larger amplitude subthreshold V_m_ responses in novel environments. (**A**) Example cell recorded in a NOV-FAM maze pair with a sufficient number of spikes for comparison of AP threshold (see Materials and methods). AP threshold versus the pre-spike subthreshold V_m_ (1000-to-50 ms before the AP) plotted for all isolated APs and/or first spikes in a high-frequency burst (excluding APs in CSs and those with shoulders) in the NOV and FAM maze. Insets: Overall epoch peak AP rates from the maze pairs. (**B**)-(**E**) Results using different methods of measuring baseline V_m_ and related quantities. (**B**) Mean of the baseline subthreshold V_m_ values during individual laps in each maze epoch for NOV-FAM maze pairs. (**C**) Overall epoch absolute median subthreshold V_m_ for all cells recorded in a NOV-FAM maze pair. (**D**) Same as (**C**) for baseline-subtracted median subthreshold V_m_. (**E**) Baseline subthreshold V_m_ (bottom 10%-ile ± SE; subthreshold V_m_ values from 10 s sliding windows, 1 s sliding steps) in NOV-FAM maze pairs around maze transitions (t = 0). Top: FAM-to-NOV transitions; Bottom: NOV-to-FAM transitions. Horizontal lines indicate the significance of paired comparisons between activity after maze transition and from −60 to 0 s before maze transition; thick lines: p<0.05, thin lines: n.s. (**F**) Difference of overall epoch baseline subthreshold V_m_ versus peak subthreshold V_m_ responses for all NOV-FAM maze pairs. (**G**) Difference of mean somatic input resistance versus peak subthreshold V_m_ responses for all NOV-FAM maze pairs. **DOI:** http://dx.doi.org/10.7554/eLife.23040.010

Together, these results indicate that hippocampal CA1 neurons responded uniquely to novelty with enhanced inputs, leading to higher firing rates than in familiar environments. Another likely contributor to the increased spiking in novel environments was larger temporal fluctuations of the subthreshold V_m_, reflecting the instantaneous input activity about the mean hill amplitudes described so far. Single place field traversals in novel and familiar mazes could display robust theta (~5–10 Hz) fluctuations (Figure 3—figure supplement 2A). As in freely moving rats (Lee et al., 2012), the theta-band power was slightly positively correlated with mean subthreshold V_m_ (Figure 3—figure supplement 2B), implying that peak theta power should be somewhat higher in novel environments. Indeed, we found slightly increased in-field theta power (5–10 Hz power^1/2^ in mV, NOV: 0.79 ± 0.09, FAM: 0.68 ± 0.06, n = 14, p<0.001), as well as gamma power (25–100 Hz power^1/2^ in mV: NOV: 0.33 ± 0.03, FAM: 0.30 ± 0.02, p=0.001), and V_m_ standard deviation (NOV: 1.31 ± 0.12 mV, FAM: 1.19 ± 0.10, p=0.011) in novel mazes (Figure 3—figure supplement 2C–E). Although small in amplitude, the extra instantaneous bump to the V_m_ hills at the peaks of these fluctuations likely adds to the enhanced spiking responses during novel exploration.

Hippocampal firing rates have been shown to increase with running speed (McNaughton et al., 1983). Here, the larger subthreshold responses in novel mazes were not attributable to differences in running behavior. Animals ran at similar overall speeds (NOV: 16.6 ± 1.3 cm/s, FAM: 15.8 ± 0.7, n = 14, p=0.63, Figure 3—figure supplement 1D) and paused (periods >1 s with speed <2 cm/s) with similar frequency (p=0.078, Figure 3—figure supplement 1E) in novel and familiar mazes. Furthermore, the instantaneous speed of the animal was not correlated with the instantaneous subthreshold V_m_ across novel and familiar epochs (median ± SE of Pearson’s r value per epoch: 0.01 ± 0.02, p=1.0). However, the differences in reward-related predictive licking (Figure 2E) and speed profile (see below) in novel versus familiar mazes provide behavioral evidence that the animals recognized the novelty and familiarity of the environments in VR.

### Somatic excitability does not contribute to enhanced responses in novel environments

We next investigated cellular properties for possible contributions to the enhanced subthreshold and spiking responses in novel mazes. Differences in postsynaptic excitability could affect the magnitude of depolarization elicited by a given presynaptic input, as well as affect the spiking response to a given subthreshold V_m_ depolarization. However, somatic excitability did not differ between novel and familiar environments according to four measures: spike threshold, baseline (resting) V_m_, input resistance, and evoked spike count (Figure 4A–D).

As already described, the AP threshold did not differ between novel and familiar environments (NOV: −47.5 ± 1.4 mV, FAM: −48.0 ± 1.7, n = 7, p=1.0, Figure 4A; also see Figure 4—figure supplement 1A). Also, as described, the baseline V_m_ – which can relate to excitability as well as to inputs – did not differ (NOV: −55.5 ± 1.3 mV, FAM: −56.6 ± 1.2, n = 14, p=0.63, Figure 4B and Figure 4—figure supplement 1B–D). A closer analysis of the baseline revealed no evidence of a role in the larger subthreshold or spiking responses during novel exploration. First, there were no transient changes in baseline found around the time of the switch between mazes (Figure 4—figure supplement 1E). In particular, there was no transient depolarization of the baseline that could trigger a sustained increase in activity during novel exploration. Furthermore, considering each neuron individually, the difference between a cell’s baseline in novel and familiar epochs could not account for the difference in peak subthreshold V_m_ (p=0.37, Figure 4—figure supplement 1F). A higher somatic membrane input resistance (R_N_) in novel mazes could cause larger subthreshold V_m_ hills in response to otherwise similar inputs. However, somatic R_N_ did not differ between novel and familiar mazes (p=0.30, Figure 4C), and any differences within individual neurons could not account for the larger peak V_m_ responses in novel environments (p=0.43, Figure 4—figure supplement 1G). Lastly, subthreshold V_m_ hills of a given amplitude could potentially result in different spiking output even with a similar AP threshold (e.g., due to an increased propensity to burst). However, the number of spikes evoked by depolarizing current steps (0.1–0.2 nA, 0.1 s) did not differ between novel and familiar environments (p=0.55, Figure 4D).

Although we found no changes in somatic excitability between novel and familiar mazes during an individual recording, we asked how intrinsic properties, such as the AP threshold, might regulate spiking activity across different recordings. In agreement with previous findings (Epsztein et al., 2011), we found that the AP threshold was negatively correlated with the mean AP rate in novel mazes (p=0.041, Figure 4E, top). Interestingly, no such correlation was found in familiar mazes, for which the mean AP rate was instead positively correlated with the baseline V_m_ (p=0.003, Figure 4F, bottom right). In addition, the baseline V_m_ was more depolarized in epochs with ≥1 AP than in epochs with no spontaneous activity for both novel and familiar mazes (NOV: p=0.002, FAM: p<0.001, Figure 4F). These results suggest that in a novel environment, intrinsic cellular excitability helps to determine the initial place field representation. Then, when the environment has become familiar, inputs can drive the stored spatial representations in CA1 irrespective of excitability.

### Spatial tuning of subthreshold V_m_ stabilizes with experience

As increases in the strength of place field inputs did not appear to be sustained, we looked for other evidence of lasting spatial learning. Stabilization of spatially tuned firing with experience is a sign of hippocampal learning (Wilson and McNaughton, 1993; Mehta et al., 1997, 2000, 2002; Kentros et al., 1998; Leutgeb et al., 2004; Frank et al., 2004; Cacucci et al., 2007). Experience-dependent changes in the spatial tuning of inputs have not previously been studied with intracellular recording. Moreover, using extracellular recording, changes cannot be inferred where there is little or no spiking, such as regions outside of place fields and in silent cells. Therefore, we analyzed the tuning of inputs across the whole environment as reflected in the shape of the entire subthreshold V_m_ curve versus location (Figure 1), and changes in this tuning with experience.

**Figure 5.**
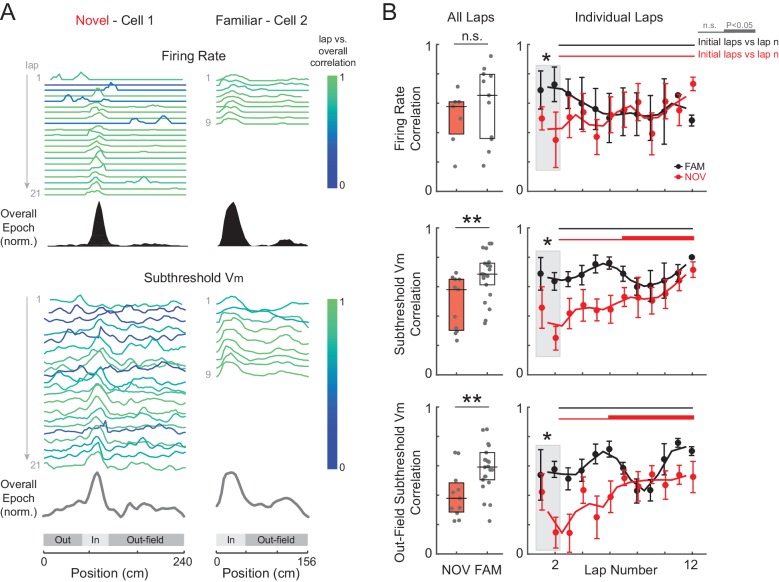
Spatial tuning of inputs throughout the entire environment stabilizes with experience. (**A**) Example of two cells recorded in a novel (left) and familiar (right) maze. AP rate (top) and subthreshold V_m_ (bottom) activity (normalized to the peak value in each lap) for each lap, color-coded based on the spatial correlation score (Pearson’s r) of the lap’s AP rate or subthreshold V_m_ profile with the overall epoch profile. (**B**) Left: Mean spatial correlation of the AP rate (top), subthreshold V_m_ in all locations (middle), and out-field (i.e., excluding the V_m_-defined field region) subthreshold V_m_ (bottom), averaged across all individual laps shown in the corresponding panels on the right. This includes all cells recorded in a NOV or FAM maze (unpaired, minimum of 4 laps required). Right: Individual lap AP rate (top), subthreshold V_m_ (middle), and out-field subthreshold V_m_ (bottom) spatial correlation scores in NOV (red) and FAM (black) mazes (mean ± SE) versus lap number. Gray box highlights the comparison of NOV versus FAM within the initial laps (1-2). Black horizontal lines indicate the significance of unpaired comparisons (two lap smoothing) between the given FAM laps and the initial laps 1–2 in the FAM mazes; thick lines: p<0.05, thin lines: n.s. Similarly, red horizontal lines are for NOV mazes. Note that (**B**) excludes all first maze epochs experienced by the animal on that day (see text and Figure 5—figure supplements 1 and 3). *p<0.05; **p<0.01. **DOI:** http://dx.doi.org/10.7554/eLife.23040.011

We assessed spatial tuning as a function of experience by computing the linear correlation of AP rate or subthreshold V_m_ activity in each lap with the average activity over the entire epoch (‘overall epoch’) (Figure 5A and Figure 5—figure supplement 1A), where each correlation was computed across all spatial bins (i.e., the entire extent of the maze). To begin with, high or low spatially correlated activity could be observed in novel or familiar mazes (Figure 5—figure supplement 2), and an individual cell’s correlation score in novel mazes did not predict its score in familiar mazes (or vice versa) for AP rate (p=0.16) or subthreshold V_m_ (p=0.83). We therefore compared spatial correlation scores for novel versus familiar epochs independent of paired exposures. All epochs were included, with the following exception. In our experiments, for each recording, the animal was generally first exposed to a familiar maze followed by a novel or other familiar maze. In many cases (10/32 cells), this first familiar maze recording was also the first exposure to any maze for the animal on that day. We suspected that spatial correlations could be disrupted in these first familiar epochs due to nonspecific behavioral factors (Figure 2—figure supplement 2B), thus partially masking differences from novel epochs. More generally, this is a factor that may be useful to consider in future VR studies. With all first epochs of the day (whether familiar or novel) excluded (Figure 5B; see Figure 5—figure supplement 1B for the same analysis including all epochs; note, excluding the initial epoch of each day did not alter any of the previous findings such as the difference in peak AP rate and subthreshold V_m_ amplitudes, Figure 5—figure supplement 3; similarly, excluding the region surrounding the primary reward location did not alter these results), we observed that AP rate correlation scores were, again, generally high and similar (p=0.29) in familiar (Pearson’s r: 0.65 ± 0.14) and novel (0.58 ± 0.09) mazes (Figure 5B, top).

**Figure 5—figure supplement 1.**
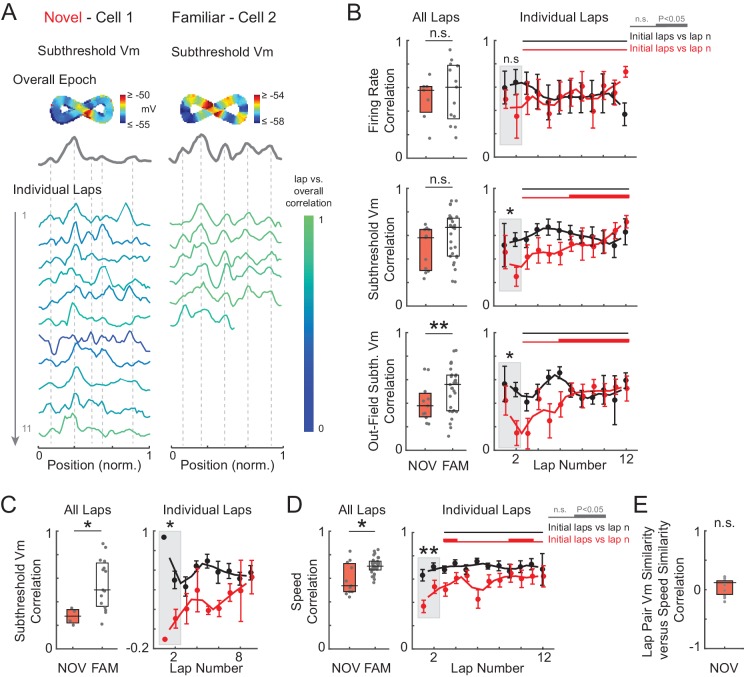
Spatial tuning as a function of experience—additional examples and analyses, including the analysis of silent cells only. (**A**) Subthreshold V_m_ spatial tuning across laps in a novel (left) and familiar (right) epoch, similar to Figure 5A. Note the increase in spatial tuning within the novel epoch, whereas the spatial correlation scores were high from the initial laps in the familiar maze. The cell is silent in this familiar epoch example. (**B**) Same analysis as Figure 5B except all maze epochs are included. Results show the same trends as Figure 5B. (**C**) Same as (B, middle panels), but for the subset of epochs in which the cell is silent. Similar to the result including all epochs, the spatial correlation scores increase within novel epochs and are high from the initial laps in familiar mazes. (**D**) Left: Same as (B, middle panels), but for running speed. (**E**) Correlation of the running speed correlation score versus the subthreshold V_m_ correlation score for all pairs of laps in NOV epochs. **DOI:** http://dx.doi.org/10.7554/eLife.23040.012

**Figure 5—figure supplement 2.**
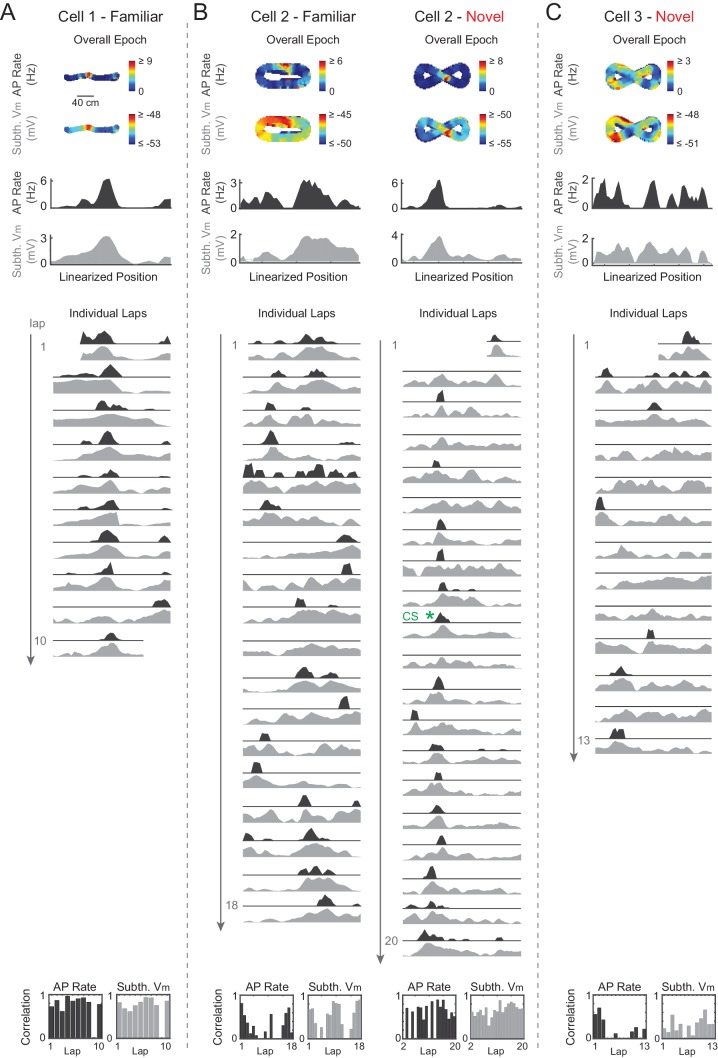
Stability of activity across laps in novel and familiar mazes, with examples of epochs that do and do not contain complex spikes. (**A**–**C**) Four example epochs. Top: Overall epoch 2-D AP rate map (shown in linearized coordinates below in black) and subthreshold V_m_ (shown in linearized coordinates below in gray); Middle: Individual lap AP rate (black) and subthreshold V_m_ (gray); Bottom: Spatial correlation scores (Pearson’s r) for the AP rate (left) and subthreshold V_m_ (right) activity of each lap versus the corresponding overall epoch activity profiles. AP rate and subthreshold V_m_ lap activity is normalized to the peak activity value on each lap. Note that Cell 2 (**B**) expresses low and high mean spatial correlation scores in two different maze epochs (FAM and NOV, respectively). Green asterisk: Place field contains a CS in that lap. Note that the novel epoch shown (**B**) is the same as that shown in Figure 6A. **DOI:** http://dx.doi.org/10.7554/eLife.23040.013

**Figure 5—figure supplement 3.**
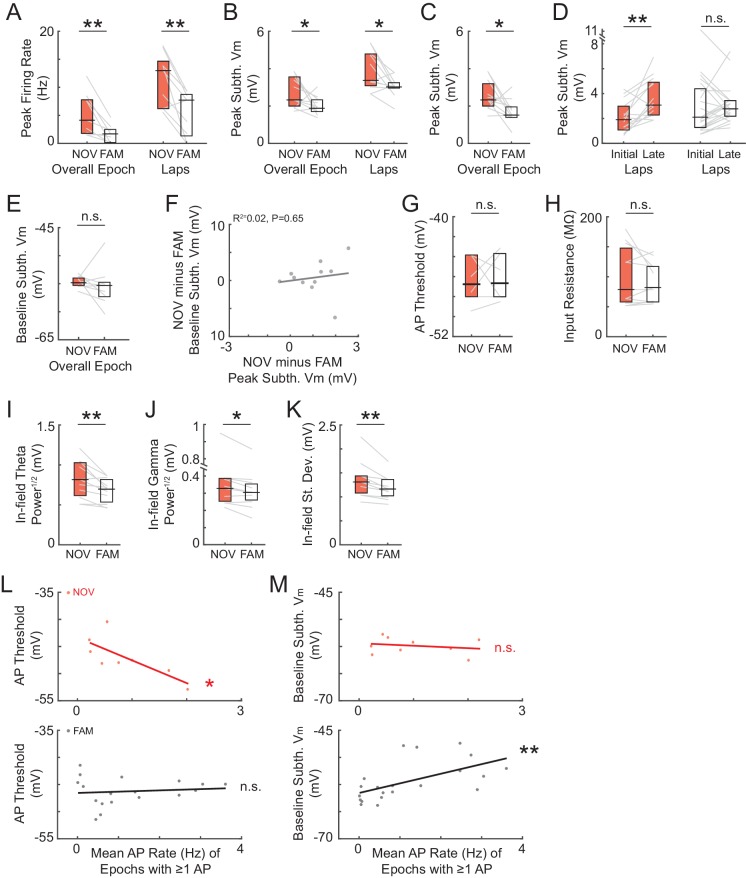
Excluding the initial maze epoch of the day does not alter the main findings of a novelty-induced enhancement of peak activity as well as other results. (**A**)-(**J**) Excluding the initial maze of the day does not alter results for NOV, FAM, and NOV versus FAM maze epoch comparisons of peak (**A**)-(**D**) (where for (B, left) and (**D**) the peak is determined within the overall epoch V_m_-defined field, and for (**C**) the peak is determined within the place field for NOV-FAM maze pairs in which there was a place field in at least one epoch of the pair) and baseline activity (**E**)-(**F**), AP threshold (**G**), input resistance (**H**), subthreshold V_m_ oscillatory power (**I**)-(**K**), or the relationship between AP threshold (**L**) and baseline subthreshold V_m_ (**M**) versus mean AP rate. *p<0.05; **p<0.01. **DOI:** http://dx.doi.org/10.7554/eLife.23040.014

**Figure 5—figure supplement 4.**
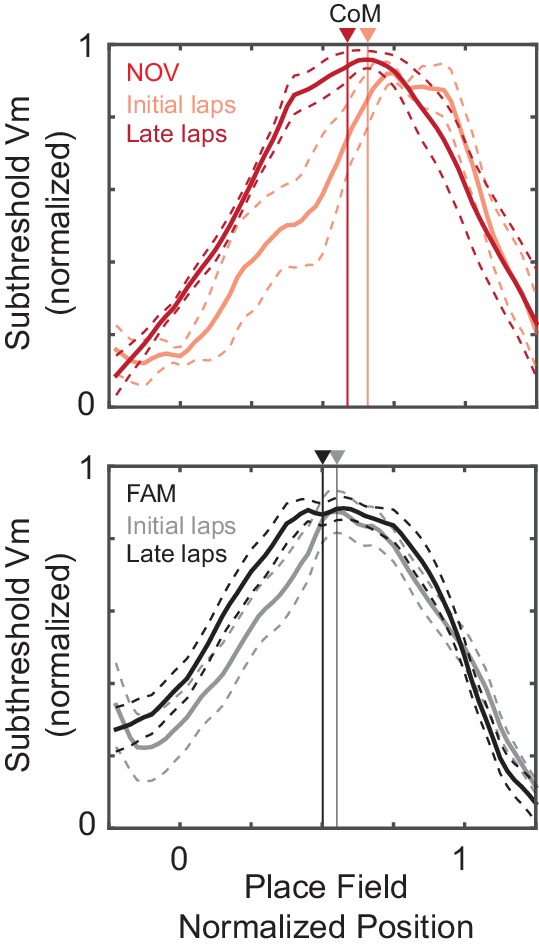
Position and shape of the subthreshold V_m_ hill as a function of experience. Normalized subthreshold V_m_ (mean ± SE) for the initial laps 1–2 and late laps 6-end with respect to the normalized position of the overall epoch AP rate field (0–1) for NOV and FAM maze epochs. Center-of-mass (CoM) of each curve marked with vertical lines. Animal running direction from left to right. Note the leftward shift (the direction opposite to the animal’s movement) of the CoM from the initial to late laps within both NOV and FAM epochs, as well as from NOV to FAM epochs. **DOI:** http://dx.doi.org/10.7554/eLife.23040.015

In contrast, the subthreshold V_m_ spatial correlation scores were significantly lower in novel than familiar mazes (NOV: 0.58 ± 0.12, FAM: 0.69 ± 0.03, p=0.003, Figure 5B, middle left). Moreover, the V_m_ correlation scores were lower in the initial laps (p=0.003, Figure 5B, middle right), then significantly increased across laps within a novel but not familiar epoch, reaching close to the familiar values in later laps (Figure 5B, middle right). This progressive stabilization of spatial tuning with experience is an intracellular signature of the establishment of a long-term memory trace.

### Spatial tuning of subthreshold V_m_ stabilizes across the entire environment

We then asked where this progressive stabilization was occurring. Extracellular studies have shown that CA1 place field spiking stabilizes with experience (Wilson and McNaughton, 1993; Frank et al., 2004; Leutgeb et al., 2004; Cacucci et al., 2007). Is the stabilization of spatial tuning we observed in CA1 occurring for its inputs across the whole environment?

We addressed this question by analyzing the subthreshold V_m_ outside of the peak subthreshold V_m_ region (‘out-field’). As with the overall spatial correlation scores, the out-field scores started low in the initial laps (p=0.008) of novel environments then increased in the later laps, and in familiar mazes were generally well-correlated across laps (Figure 5B, bottom). Furthermore, for the subset of epochs in which cells were silent, the spatial correlation of the subthreshold V_m_ also increased during novel exploration and was high in familiar environments (Figure 5—figure supplement 1C). These results indicate that the increase in spatial tuning was not limited to the place field region, and that the spatial tuning of the rest of the inputs also stabilizes. This is consistent with stabilization of the entire network with experience, including excitatory inputs from hippocampal area CA3 and the entorhinal cortex (EC), each of which contain neurons that display spatially tuned firing (e.g., place fields in CA3 and grid cells in EC) that must itself be established in novel environments (Leutgeb et al., 2004; Barry et al., 2012).

While animals ran at similar speeds in novel and familiar environments, the spatial profile of running speed was somewhat less consistent during novel exploration (p=0.025; Figure 5—figure supplement 1D). However, the lower V_m_ correlation scores in novel mazes were not attributable to differences in running behavior, as speed correlation scores did not predict V_m_ correlation scores (p=0.28; Figure 5—figure supplement 1E; see Materials and methods).

We also measured the shape of the subthreshold V_m_ hills. A backward shift of the hill with experience (i.e., a shift in location in the direction opposite to the animal’s motion) (Blum and Abbott, 1996; Mehta et al., 1997) and a negative skew (i.e., a slower rise in the direction of the animal’s motion) (Mehta et al., 2000; 2002; Ekstrom et al., 2001; Harvey et al., 2009) are thought to result from spike timing-dependent plasticity (Markram et al., 1997; Bi and Poo, 1998). Signs of both were observed in the subthreshold V_m_ hills underlying the AP place field (Figure 5—figure supplement 4), providing additional evidence of experience-dependent plasticity.

### Complex spikes are not necessary for place field formation in novel environments

**Figure 6.**
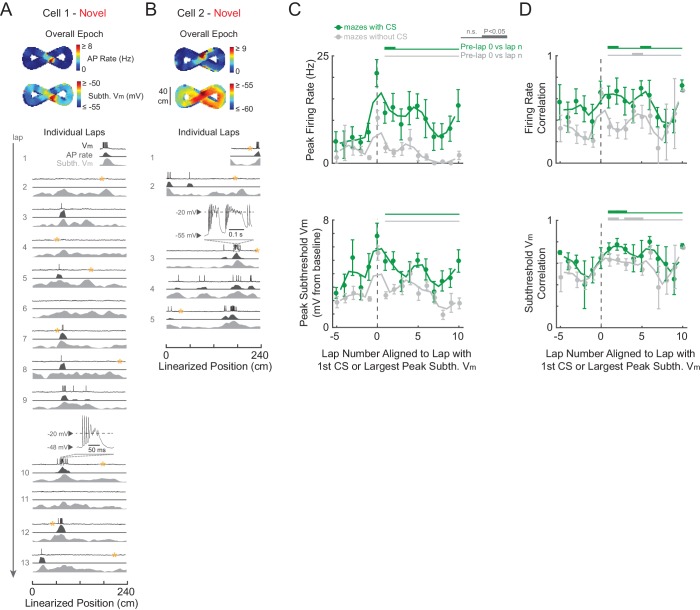
Complex spikes transiently boost spatial responses but are not required for place field formation in novel mazes. (**A**)-(**B**) Example novel maze recordings that contained ≥1 CS in the maze. Overall epoch 2-D AP rate map and subthreshold V_m_ (above). Individual laps are shown as three traces: V_m_ (top), AP rate (middle), and subthreshold V_m_ (bottom), with the location and trace of the first CSs in each maze epoch highlighted. AP rate and subthreshold V_m_ lap activity is normalized to the peak activity value in each lap. In no cases were CSs evoked by the current injections (asterisks) in (**A**)-(**B**). In (**A**), the place field had already formed before the first CS occurs. In (**B**), the CS occurs in the lap in which the place field first appears (same epoch as shown in Figure 3C). Note that there were no CSs in the novel maze place field recordings in Figure 2B or Figure 3D. (**C**) Peak AP rate (top) and subthreshold V_m_ (bottom) for individual laps aligned (lap = 0) to the lap containing the first CS in the maze epoch for those epochs with a CS (green) or containing the largest peak subthreshold V_m_ for those epochs without a CS (gray), then pooled across NOV and FAM maze epochs. Peak activity determined from the region (±3 spatial bins) surrounding the CS or peak subthreshold V_m_ location. Mean ± SE. Green horizontal lines indicate the significance of paired comparisons (two lap smoothing) between the given laps and the mean of the pre-CS (pre-lap 0) laps; thick lines: p<0.05, thin lines: n.s. Similarly, gray horizontal lines are for epochs without a CS. (**D**) Same as (**C**) for AP rate (top) and subthreshold V_m_ (bottom) spatial correlation scores (Pearson’s r). **DOI:** http://dx.doi.org/10.7554/eLife.23040.016

Finally, we examined the role of CSs in place field formation and plasticity during novel environment exploration. CSs (also called plateau potentials in Bittner et al., 2015) are detected in somatic recordings as large amplitude, plateau-like V_m_ depolarizations with accompanying spiking (Figure 6A–B, expanded traces), and are distinct (Epsztein et al., 2011) from extracellularly defined complex spikes (Ranck, 1973). CSs occurred in novel and familiar mazes and had similar properties in both (e.g., duration, number of APs, Figure 6—figure supplement 1A–C). CSs spontaneously occurred at low rates in both novel (14 CSs total in 10 of 190 total laps from nine maze epochs with ≥1 AP) and familiar (17 CSs in 11/228 laps from 26 epochs) mazes, were present in less than half of all cells (Figure 6—figure supplement 1D, left), and had occurrence rates that increased similarly with increasing subthreshold V_m_ level (Figure 6—figure supplement 1E).

**Figure 6—figure supplement 1.**
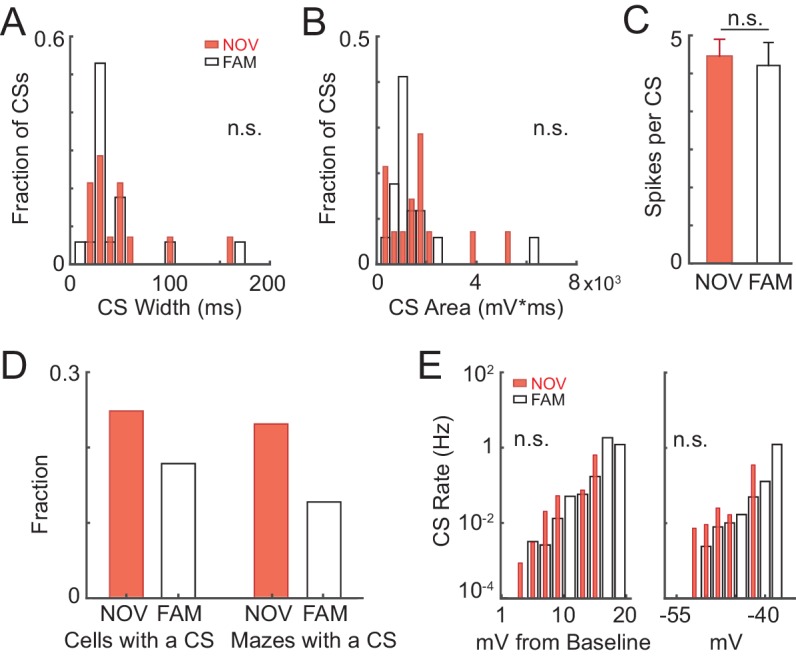
Properties of intracellular complex spikes in novel and familiar mazes. (**A**)-(**C**) CS width (at half-maximum, ms) (**A**), CS area (mV*ms) (**B**), and number of spikes per CS (**C**) (mean ± SE) for all CSs in NOV and FAM mazes. (**D**) Left: Fraction of cells recorded in NOV and FAM mazes that had at least one CS in a NOV or FAM maze. Right: Fraction of all NOV and FAM maze epochs in which at least one CS occurred in the maze. (**E**) CS rate as a function of relative (to lap baseline, left) and absolute (right) subthreshold V_m_. Here, subthreshold V_m_ is computed as the mean value in 150 ms windows (sliding every 10 ms). **DOI:** http://dx.doi.org/10.7554/eLife.23040.017

**Figure 6—figure supplement 2.**
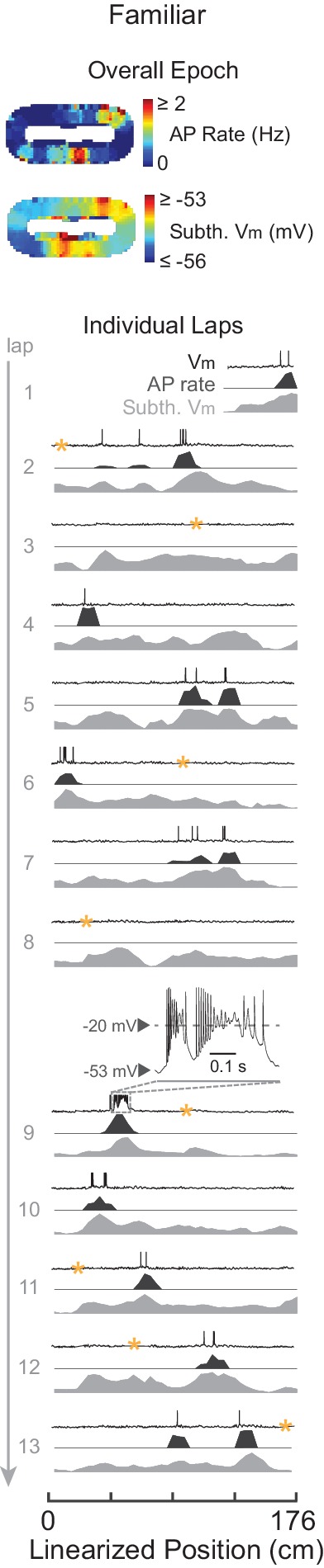
Complex spikes in a familiar maze. Example familiar maze recording that contained ≥1 CS in the maze. Overall epoch 2-D AP rate map and subthreshold V_m_ (above). Individual laps are shown as three traces: V_m_ (top), AP rate (middle), and subthreshold V_m_ (bottom), with the location and trace of the first CS in the maze epoch highlighted. AP rate and subthreshold V_m_ lap activity is normalized to the peak activity value in each lap. In this example, the CS did not stabilize the location of the place field. CSs occurred in five other familiar epochs (in four cases spiking activity was already stable before the CS, and in the other case the CS occurred in the first lap that the place field appeared). **DOI:** http://dx.doi.org/10.7554/eLife.23040.018

Even though CSs were rare events, previous work suggests that they play a significant role in the formation of place fields in novel mazes, as has been shown for familiar environments. Specifically, in previous work using a familiar treadmill-based virtual environment, CSs were always present in the first lap of spontaneously appearing place fields in cells that were silent in previous laps, and artificially evoked CSs were capable of immediately creating place fields in previously silent cells (Bittner et al., 2015). Therefore, we checked whether the initial appearance of place fields in novel mazes, which by definition are new place fields, was accompanied by CSs. Of the nine novel epoch place fields, four displayed stable activity in the place field region prior to any CSs (which then occurred later in the place field in 3/4 cases, e.g., Figure 6A), two had ≥1 CS in the initial lap of the emergent field (e.g., Figures 3C and 6B), and the remaining 3 were in epochs that did not have any CSs (e.g., Figures 2B and 3D did not contain CSs). Of these 9 place fields, 7 occurred in mazes that the animal had never previously been exposed to, and 2 occurred in mazes previously experienced 1 or 2 times (versus ≥19 exposures for familiar epochs, Table 1). Thus, CSs were not required for place field formation in novel environments. Moreover, CSs in familiar mazes did not necessarily stabilize place fields (Figure 6—figure supplement 2).

**Table 1.** Details of the individual cells and maze epochs. The ‘P’ and ‘N’ for epochs in mazes which the animal explored in both directions denote the two directions (i.e. moving in the direction of positive and negative changes in position value). The ‘#” indicates epochs that were excluded from analysis because their place fields appear to have been attributable to experimenter manipulations (e.g. inadvertent triggering of a CS during a current step used to probe series and input resistance and evoked spiking response), and thus their place field properties could not be assumed to be those of spontaneously occurring place fields. These epochs were however included in the analysis of Figure 6C–D since one can still assess the effect of any spontaneous CSs they displayed. The ‘##” marks an epoch in which the AP threshold could not be measured because all APs in the epoch contained a shoulder (see Materials and methods). **DOI:** http://dx.doi.org/10.7554/eLife.23040.019

**Cell #**	**Epoch #**	**Maze**	**# Prev. Maze Sessions**	**Rec. dur. (s)**	**# Laps**	**Subth. vm baseline (mV)**	**Subth. vm peak (mV)**	**AP rate peak (Hz)**	**AP rate mean (Hz)**	**AP threshold (mV)**	**Input R. (MOhm)**	**AP rate Infield-Outfield ratio**	**Place field (0=No, 1=Yes)**
1	1	'O'	≥39	106.5	7	−61.3	1.4	0.00	0.00	N/A	147.5	N/A	N/A
1	2	'8'	0	282.7	17	−60.3	2.4	0.00	0.00	N/A	197.0	N/A	N/A
2	1	'O'	≥39	99.7	5	−56.9	1.2	0.00	0.00	N/A	88.8	N/A	N/A
2	3P	'l'	≥19	209.0	3	−54.0	2.8	0.00	0.00	N/A	N/A	N/A	N/A
2	3N	'l'	≥19		2	−53.7	2.1	0.00	0.00	N/A	N/A	N/A	N/A
3	1	'O'	≥39	171.5	6	−56.5	1.9	2.32	0.59	−48.5	77.7	6.1	1
3	2	'8'	1	124.3	5	−55.4	1.7	4.14	1.68	−49.4	195.3	1.7	0
4	2	'8'	0	807.7	13	−52.8	1.1	1.35	0.44	−48.1	58.1	2.1	0
5	1	'O'	≥39	115.5	5	−62.6	1.3	0.00	0.00	N/A	120.3	N/A	N/A
6	1	'O'	≥39	103.1	8	−57.2	2.0	0.00	0.00	N/A	83.6	N/A	N/A
6	2P	'L'	0	587.7	12	−53.3	2.3	2.67	0.78	−40.4	23.9	4.5	1
6	2N	'L'	0		12	−53.6	1.7	0.89	0.38	−40.4	23.9	7.1	1
7	1	'O'	≥39	96.6	8	−66.1	1.4	0.00	0.00	N/A	83.4	N/A	N/A
7	2	'8'	1	147.7	9	−66.4	1.9	0.00	0.00	N/A	86.8	N/A	N/A
7	3P	'l'	≥19	124.4	5	−66.6	3.0	0.00	0.00	N/A	69.0	N/A	N/A
7	3N	'l'	≥19		5	−65.6	2.0	0.00	0.00	N/A	69.0	N/A	N/A
8	1	'O'	≥39	174.9	6	−54.7	1.6	0.80	0.07	N/A	N/A	32.6	0
9	2	'8'	0	121.0	6	−59.4	1.1	0.00	0.00	N/A	68.9	N/A	N/A
10	1	'O'	≥39	143.9	5	−57.0	2.0	6.58	1.36	N/A	128.0	3.9	1
11	1	'O'	≥39	57.5	6	−57.8	1.1	0.00	0.00	N/A	117.3	N/A	N/A
11	2#	'8'	0	1058.3	9	−55.7	7.9	12.05	0.89	−42.2	112.7	35.5	1
12	1	'O'	≥39	248.1	16	−55.3	2.2	2.89	1.51	−47.3	83.8	14.5	1
12	2P	'l'	≥19	67.5	4	−55.9	3.9	9.52	2.57	−46.0	73.6	3.7	1
12	2N	'l'	≥19		3	−55.6	3.4	14.08	4.14	−46.0	73.6	3.3	1
13	1	'8'	0	1012.1	27	−57.5	3.3	11.26	2.02	−52.8	145.1	6.7	1
14	1	'O'	≥39	224.8	13	−54.9	1.7	2.10	0.80	N/A	69.0	1.5	0
14	2	'8'	1	367.1	21	−55.6	3.6	8.46	0.75	−48.0	55.4	25.7	1
14	3	'O'	≥39	250.2	19	−55.4	1.7	1.69	0.45	−48.0	45.1	3.3	1
14	4	'8'	2	428.4	25	−55.0	2.3	1.49	0.22	−43.8	55.3	6.6	1
14	5P	'l'	≥19	203.5	9	−53.9	2.2	0.22	0.02	−45.3	100.9	20.0	0
14	5N	'l'	≥19		9	−53.9	1.7	2.31	0.22	−45.3	100.9	34.3	1
15	1	'O'	≥39	37.6	4	−60.5	1.5	0.00	0.00	N/A	40.4	N/A	N/A
15	2	'8'	0	505.6	26	−54.2	3.8	7.56	1.00	−47.5	49.9	15.3	1
15	3	'O'	≥39	199.5	18	−47.8	1.9	3.02	1.08	−43.7	57.4	3.0	1
16	1	'O'	≥39	99.7	6	−58.8	2.5	0.50	0.04	N/A	59.8	29.4	0
16	2#	'8'	1	302.6	20	−57.0	3.2	8.86	2.48	−50.4	56.8	3.2	1
16	3	'O'	≥39	198.2	8	−58.8	3.9	3.79	0.45	−51.5	49.9	13.9	1
16	4#	'8'	2	271.3	13	−54.3	4.6	14.51	4.06	−50.7	39.9	5.0	1
16	5	'O'	≥39	306.0	7	−57.4	3.0	2.67	0.55	−50.6	N/A	5.5	1
16	6#	'8'	3	421.1	17	−61.5	2.9	3.45	0.42	N/A	N/A	23.3	1
17	1P	'l'	≥19	604.2	9	−52.5	6.6	7.99	1.53	−47.0	42.6	7.5	1
17	1N	'l'	≥19		10	−52.3	3.6	21.46	4.64	−47.0	42.6	11.4	1
18	1	'O'	≥39	47.3	4	−61.2	1.7	0.00	0.00	N/A	N/A	N/A	N/A
19	1	'O'	≥39	191.9	4	−56.7	2.6	0.48	0.03	N/A	N/A	Inf	0
19	2	'8'	0	221.2	3	−64.3	1.6	0.00	0.00	N/A	N/A	N/A	N/A
20	1	'O'	≥39	154.7	9	−61.6	1.2	0.00	0.00	N/A	140.6	N/A	N/A
20	2	'8'	≥19	65.2	3	−63.8	3.0	0.00	0.00	N/A	196.1	N/A	N/A
21	1	'O'	≥39	169.6	10	−54.9	0.8	0.54	0.04	N/A	169.4	79.0	0
21	2	'8'	≥19	60.1	3	−53.1	1.0	0.00	0.00	N/A	N/A	N/A	N/A
22	1	'O'	≥39	149.2	9	−57.2	1.6	0.23	0.01	−44.6	76.8	Inf	0
22	2	'8'	≥19	137.9	5	−55.6	1.7	0.00	0.00	N/A	N/A	N/A	N/A
23	1	'O'	≥39	233.1	5	−63.1	3.0	0.00	0.00	N/A	N/A	N/A	N/A
23	2	'8'	≥19	206.1	4	−47.4	2.3	7.49	2.47	−44.3	183.5	3.1	1
24	1	'O'	≥39	295.6	8	−48.2	1.6	3.96	1.41	−46.4	159.3	2.7	0
24	2	'8'	≥19	384.7	5	−49.6	5.8	14.50	2.73	N/A	N/A	5.4	1
25	1	'O'	≥39	175.8	9	−58.1	2.4	1.17	0.07	−43.2	85.6	Inf	0
25	3P	'L'	0	1214.3	10	−56.4	1.2	0.09	0.01	−46.0	80.8	Inf	0
25	3N	'L'	0		11	−56.5	3.4	1.90	0.46	−46.0	80.8	31.5	0
26	1	'O'	≥39	53.2	3	−60.6	2.5	0.00	0.00	N/A	N/A	N/A	N/A
27	1	'O'	≥39	77.9	4	−52.0	8.4	12.61	3.61	−44.9	77.4	3.6	1
27	2	'8'	≥19	399.4	4	−53.5	8.2	14.84	3.06	−44.9	N/A	4.0	1
27	3	'O'	≥39	105.2	8	−56.4	3.8	4.26	0.81	−46.6	67.1	4.3	1
27	4	'8'	≥19	112.8	6	−57.8	3.9	0.57	0.03	N/A	N/A	Inf	0
28	2	'8'	≥19	217.2	4	−55.4	2.5	0.76	0.06	−41.4	145.7	23.3	1
29	1	'O'	≥39	135.3	7	−59.4	1.7	0.00	0.00	N/A	75.4	N/A	N/A
30	1	'O'	≥39	141.7	6	−60.9	1.2	0.00	0.00	N/A	43.4	N/A	N/A
31	2	'8'	≥19	82.4	3	−56.0	1.5	0.00	0.00	N/A	N/A	N/A	N/A
32	2	'8'	≥19	159.9	5	−55.2	2.5	4.66	0.82	−48.3	124.4	4.6	1
32	3P	'L'	0	378.1	14	−53.8	3.0	11.95	3.29	N/A##	145.1	3.1	1
32	3N	'L'	0		14	−53.7	2.7	5.43	1.55	N/A##	145.1	3.1	1

In addition to a specific role in forming new place fields in familiar environments (Bittner et al., 2015), CSs have been shown to trigger synaptic plasticity in general (Takahashi and Magee, 2009), so we examined the possibility that they contribute to the experience-dependent changes in activity that we observed. We checked whether CSs may contribute to the stabilization of activity by boosting subsequent activity and spatial correlation scores. We aligned each lap’s activity to the lap containing the first CS in each epoch (whether novel or familiar). For comparison to epochs that did not contain CSs, we aligned laps in those epochs to the lap with the largest peak subthreshold V_m_ (since CSs tend to occur at the most depolarized V_m_ values, Figure 6—figure supplement 1E). We found a transient, but significant, increase in AP rate in the first lap after the first CS (Figure 6C, top), but not in subsequent peak subthreshold V_m_ activity (Figure 6C, bottom). Regarding spatial tuning, we found a transient increase in AP rate and subthreshold V_m_ spatial correlation scores after the first CS, though a similar result for the subthreshold V_m_ spatial correlation was found following the largest peak V_m_ for maze epochs without CSs (Figure 6D). Therefore, individual CSs appear to contribute an amount to plasticity and spatial learning that varies in magnitude – from small (i.e., transient increases in spiking and spatial correlation) to potentially large (i.e., occurring in the first lap of new place fields).

## Discussion

**Figure 7.**
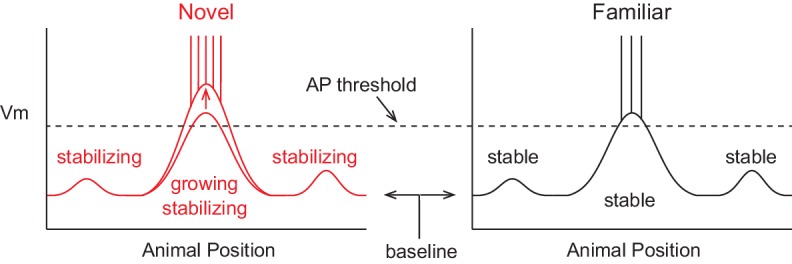
Summary of intracellular features underlying the formation of hippocampal CA1 place cell representations. Schematic based on findings from this study (compare to Figure 1). During novel exploration (left), the subthreshold V_m_ depolarization underlying place fields grows in amplitude. This increase in amplitude is not sustained as the environment becomes more familiar (right). However, the spatial tuning of inputs, which is initially lower in all locations of novel mazes, stabilizes during novel exploration, both inside and outside of the place field. The higher AP rate in novel environments is due to the larger amplitude subthreshold depolarizations, as no differences were observed in the baseline V_m_ or somatic excitability (e.g., AP threshold). CSs occur in a fraction of novel maze epochs and the appearance of place fields in novel environments does not require CSs. Thus, stable representations of familiar mazes are supported by the emergence of less strongly modulated, but more repeatable, spatially tuned subthreshold inputs. **DOI:** http://dx.doi.org/10.7554/eLife.23040.020

Using intracellular recording, we have investigated the mechanisms underlying how hippocampal spatial representations form and stabilize with experience. We began by testing a model of this process that was based on previous experimental and theoretical work (Figure 1). In summary (Figure 7), we found that the inputs driving place field spiking did indeed increase in amplitude during the exploration of a novel environment (Figure 3). Furthermore, this growth could occur before spiking occurred, suggesting a role for plasticity mechanisms that do not depend on postsynaptic spiking (Golding et al., 2002; Dudman et al., 2007). However, these increases in input amplitude were not sustained after additional experience in the environment, suggesting the need for alternate mechanisms of establishing stable place field activity in familiar environments. We then found an experience-dependent change that was maintained with familiarization – an increase in the similarity of the spatial tuning of inputs across the entire extent of the environment (Figure 5). This is a signature of the long-term stabilization of the hippocampal representation and provides a foundation for reliable spatial firing in familiar environments.

We found that complex spikes (CSs), which can induce synaptic plasticity (Takahashi and Magee, 2009) and are sufficient to trigger place field formation in familiar environments (Bittner et al., 2015), were not required for the appearance of individual place fields during novel exploration (e.g., Figures 2B, 3D and 6A). However, temporary increases in firing rate were seen in the laps subsequent to CS occurrence (Figure 6C). This suggests that CS-based plasticity could contribute to the long-term stabilization of place fields, perhaps in a similar fashion to other large, calcium-mediated events (Sheffield and Dombeck, 2015). This would be consistent with experiments in which NMDAR-dependent plasticity is not necessary for the formation of place fields in novel environments (McHugh et al., 1996; Kentros et al., 1998), but rather for their persistence across days (Kentros et al., 1998). Note that CSs could be more potent triggers for place field formation in proximal cue-based environments (Bittner et al., 2015) versus the distal cue-based environments of our study.

While inputs and spiking were boosted during novel exploration, we found no differences in the baseline V_m_ and somatic excitability between novel and familiar environments (Figure 4A–D and Figure 4—figure supplement 1). However, across different cells recorded in novel environments, firing rates were inversely correlated with AP threshold (Figure 4E), in agreement with previous work showing that cells with higher excitability are more likely to display place field activity during novel exploration (Epsztein et al., 2011; Rich et al., 2014).

Altogether, our findings support the following model of hippocampal spatial learning: (1) initially, small inhomogeneities in spatial inputs and excitability present during the first experience in an environment are amplified to produce robust place field activity without the need for plasticity; (2) plasticity during repeated traversals of the environment progressively refines and stabilizes this representation for long-term storage; (3) after learning, the inputs are no longer amplified, but the stability of these less strongly modulated inputs yields reliable spatially tuned firing. That is, the role of plasticity is to capture the initial representation and stabilize it before the network changes again, perhaps as a result of learning in other contexts in intervening periods. Plasticity would presumably stabilize CA1 place fields that happened to receive sufficiently strong, consistent, and/or matched (e.g., both EC and CA3) inputs (Dudman et al., 2007; Sheffield and Dombeck, 2015; Bittner et al., 2015; Basu et al., 2016). The initial expansion of activity (i.e., greater numbers of more active cells) could provide a diversity of synaptic and cellular patterns from which a sparser representation of space can be selected for long-term stabilization. The amplification of spiking activity could also serve to drive plasticity in downstream targets (Lisman, 1997; Harris et al., 2001; Xu et al., 2012) or send a general novelty signal (Duncan et al., 2012; Larkin et al., 2014) to the rest of the brain, which in turn may drive neuromodulatory feedback to the hippocampus and promote learning (Lisman and Grace, 2005). Afterwards, the comparatively low amplitude of inputs and firing rate in familiar environments may function to limit changes to the representation, further contributing to its stability. Additional changes, such as the creation of new fields via CSs (Bittner et al., 2015) at behaviorally important locations (Hollup et al., 2001; Dupret et al., 2010; Monaco et al., 2014), would presumably occur within a now stable spatial context. Variability of the low amplitude, but spatially reliable, modulation of inputs across the environment could explain why a given place cell is not always active in the same environment, but that when it fires, it does so in the same location (Ziv et al., 2013; Rubin et al., 2015).

What might underlie the amplification of inputs during novel spatial exploration? To begin with, there could be increased activity in input areas. However, the evidence to date indicates no difference in the average firing rates of excitatory neurons in CA3 (Karlsson and Frank, 2008) or EC (Barry et al., 2012) in novel compared to familiar environments. Whether there are differences in the activity of other excitatory inputs to CA1, such as the nucleus reuniens (Herkenham, 1978; Vertes, 2015), frontal cortex (Rajasethupathy et al., 2015), or the amygdala (Sheth et al., 2008) is currently unknown.

There are also multiple means by which the same excitatory input activity could result in larger amplitude depolarizations. Differences in the relative timing of inputs within and/or between, for example, CA3 and EC (Dudman et al., 2007; Penley et al., 2013; Bittner et al., 2015) could boost integration. The larger subthreshold theta oscillations we observed could reflect such altered timing, but could also simply be due to the increased peak V_m_ values in novel mazes combined with the observed relationship between intracellular theta power and V_m_ (Kamondi et al., 1998; Lee et al., 2012) (which could be caused by an increased driving force with respect to the inhibitory reversal potential). In addition, altered timing alone would not necessarily lead to the larger peak subthreshold responses we measured, as they involved the mean V_m_ over many theta cycles.

Several types of changes in excitability could increase the size of the subthreshold response to a given input. First, a more depolarized somatic baseline V_m_ can greatly enhance the subthreshold response of CA1 pyramidal neurons to spatial inputs (Lee et al., 2012, 2014). However, as mentioned above, we found no evidence for differences in baseline V_m_ (or input resistance) that could account for the larger peak subthreshold V_m_ responses in novel mazes. While we did not detect differences in somatic excitability, changes in inhibition (Lovett-Barron et al., 2012; Royer et al., 2012; Milstein et al., 2015) or intrinsic conductances (Johnston et al., 2003; Johnston and Narayanan, 2008; Giessel and Sabatini, 2010) in dendrites could alter the response to synaptic inputs. Neuromodulators such as acetylcholine (Hasselmo, 2006; Teles-Grilo Ruivo and Mellor, 2013) and dopamine (Li et al., 2003; Kentros et al., 2004; McNamara et al., 2014; Takeuchi et al., 2016) could underlie a novelty-specific enhancement of inputs. For instance, neuromodulators have been shown to affect the gain of receptive field responses (Fu et al., 2014). An analogous mechanism could be at work here, though with a difference; the gain change shown in recent work occurs when comparing active and quiescent behavior (Chiappe et al., 2010; Maimon et al., 2010; Niell and Stryker, 2010; Bennett et al., 2013; Polack et al., 2013; Fu et al., 2014; Schneider et al., 2014). In our case, the difference in responsiveness would occur between two similar active behaviors – running at similar speeds in environments differentiated only by the degree of familiarity. On the other hand, unlike what could be expected from an overall change in gain, we did not observe a difference in the amplitude of the second highest subthreshold V_m_ peaks in novel compared to familiar environments.

Instead of an amplification of inputs in novel environments, alternate mechanisms such as habituation or synaptic depression (Xu et al., 1998; Kemp and Manahan-Vaughan, 2007) could decrease response magnitudes across days (Lim et al., 2015). In such models (Lim et al., 2015), the larger response to novel versus familiar items occurs in a bottom-up fashion and thus could occur without a novelty-specific signal. In any case, the increasing lap-to-lap similarity of the subthreshold V_m_ indicates that some form of plasticity underlies the formation of stable representations of familiar environments.

The correlation between spike rate and AP threshold in novel but not familiar environments (Figure 4E) is consistent with a model in which initial differences in intrinsic excitability bias which cells will be originally recruited into a given memory representation, with changes in excitability over time resulting in the allocation of different sets of cells to subsequent experiences (Han et al., 2007; Zhou et al., 2009; Epsztein et al., 2011; Cai et al., 2016; Rashid et al., 2016). In particular, the lack of correlation between firing rate and threshold in familiar environments suggests that, after the stabilization of a representation, inputs determine activity irrespective of excitability levels. Furthermore, the correlation between firing rate and baseline V_m_ in familiar environments (Figure 4F) suggests that learning leads to the emergence of a cell assembly (Hebb, 1949) representing each maze, resulting in a spatially uniform subthreshold depolarizing bias for all place cells active in a given environment. In this case, the lower amplitude of the V_m_ hills under place fields in familiar mazes may not represent weaker inputs. Rather, the inputs driving place fields may have remained strengthened, but the out-of-field inputs may also have strengthened with familiarization, thus reducing the amplitude of the hill relative to the baseline. Meanwhile, the higher firing rate of inhibitory neurons in CA1 in familiar environments (Wilson and McNaughton, 1993; Frank et al., 2004; Nitz and McNaughton, 2004) could reduce the V_m_ everywhere, keeping the average cell’s baseline V_m_ similar to that in novel environments, thereby masking the presence of these strengthened synapses.

## Materials and methods

### Virtual Reality software and behavioral setup

Our custom virtual reality software was developed at the HHMI Janelia Research Campus as part of Janelia’s open-source virtual reality software platform (Jovian). Our software suite, named ‘MouseoVeR’, is written in C++ and built from a number of open-source software components (Boost, Bullet, osgBullet, osgWorks, OpenSceneGraph, Collada, OpenGL, and Qt) that work together to generate a system allowing users to create configurable visual environments. The MouseoVeR code used in this study is available at https://github.com/JaneliaSciComp/CohenBolstadLee_eLife2017. Virtual maze environments were created using the open-source animation software Blender (www.blender.org) and rendered by MouseoVeR. Blender environments were rendered using three virtual camera objects located at a single point in space. One camera faced forwards and captured a field of view (FOV) of 75°. The two other cameras were identical but rotated ±75° to create a total azimuthal FOV of 225°. For display, the three images were rear-projected onto a 30 in diameter, ~40% translucent cylindrical screen to create a final image encompassing a total FOV of 20° below, 60° above, and ±112.5° lateral with respect to the location of head fixation (Figure 2A, top). The cylindrical screen surrounded the spherical treadmill – a large and hollow lightweight polystyrene sphere (16 in diameter, 65 g) resting on a bed of ten individually air-cushioned ping-pong balls in an acrylic frame (http://www.flintbox.com/public/project/26501/). To track the motion of the treadmill, two cameras separated by 90° were positioned at the equator and focused on 4 mm^2^ regions under infrared light (modified from FlyFizz, https://openwiki.janelia.org/wiki/display/flyfizz/Home; Seelig et al., 2010). The cameras captured 30 × 30 pixel images of the treadmill surface at 4 kHz. Motion of the treadmill was computed from the accumulated differences in the images over time. A brief description of the MouseoVeR processing loop is as follows. In each iteration of the rendering loop, MouseoVeR communicates with the treadmill’s data server to retrieve the updated motion values since the last request. The values coming from the data server are defined as Euler angles in the server’s coordinate space. For calibration, we created mappings between 180° rotations of the treadmill along each of its three rotational axes (roll, pitch, and yaw) in real-world space and the data server’s coordinate space. For converting real into virtual behavior, we ignored any yaw rotations and extracted from pitch and roll values a rotational direction as well as a magnitude of rotation (which was converted into an arc length using the treadmill radius). Manually (i.e., animal-) controlled heading direction was derived from the treadmill’s pitch-to-roll ratio by continuously sampling, smoothing (~500 ms), and converting it into a °/s turning rate. The motion vector was sent to the physics engine to compute collisions with virtual objects (e.g., maze walls), and the resulting virtual movement was rendered for display at a synchronized projector frame rate of 30 Hz.

### Behavioral training

Eight-to-twelve week-old male C57BL/6NCrl (Charles River Laboratories, Wilmington, MA) mice were used for all experiments. All procedures were performed in accordance with the Janelia Research Campus Institutional Animal Care and Use Committee guidelines on animal welfare. Prior to behavioral training, a stainless steel head plate – with a large central recording well to access the hippocampus bilaterally – was attached to the skull surface using light-cured adhesives (Optibond All-in-One, Kerr; Charisma, Heraeus Kulzer, South Bend, IN) and dental acrylic. Sites for future hippocampal CA1 craniotomies (bregma: −1.6 to −2.0 mm AP, 1.2 to 1.8 mm ML) were marked with a fine-tipped cautery pen (Medline Inc., Northfield, IL). Mice were allowed to recover for at least five days before behavioral training started. During recovery, the mice were given food and water ad libitum, and a saucer running wheel was placed in the home cage.

The day before starting behavioral training, mice were placed on water-restriction (1.0 ml/day). From this day onwards, the saucer wheel was removed from the home cage overnight. Body weight and overall health were checked each day to ensure the mice remained healthy over the course of the experiment (Guo et al., 2014). On day 1, mice were acclimated to the experimental setup and head fixation procedure over several short sessions (increasing from ~1 to 15 min). The head was centered and fixed atop the treadmill, with the eyes ~20 mm from the surface. This created a comfortable ~20° angle below horizontal between the skull surface and the neck-to-tail body line. A tube (2.0 mm O.D. thick-walled glass with silicone-coated tip) was positioned near the mouth for delivery of artificially sweetened water rewards (acesulfame potassium, 4 mM, Sigma-Aldrich, St. Louis, MO;~2 µl liquid drop per reward). Initially, mice earned rewards by simply licking the reward tube, and then later, by producing forward motions on the treadmill of increasing distances. Mice were shown the initial virtual maze, but motion in the maze was disabled and their view was restricted to that of the start location.

On day 2, mice were exposed to the initial virtual maze for a total of ~1 hr over three training sessions (~10, 20, and 30 min durations). Four 1-D virtual mazes were used in the study (Figure 2A, middle). Two mazes were closed paths: an oval- (~175 cm length) and a figure-8-shaped track (~240 cm length). Two mazes were bidirectional: an approximately straight track (~160 cm in each direction) and an L-shaped track (~135 cm in each direction). Mice were trained to explore the virtual mazes by navigating in accordance with the geometry of the maze (e.g., heading to the right when the path veers to the right). Rewards were earned as the mice completed laps around the virtual environments. For closed path mazes, a single location was selected as the primary reward zone where two rewards were always delivered after completing a lap. In addition, ~1 reward was delivered per lap in a random location for additional motivation. For bidirectional mazes, the primary reward zone for each direction was located at the corresponding end of the track where either 1 or 2 rewards were dispensed. In addition, ~1 reward was delivered at a random location per direction. If a mouse consumed less than 1.0 ml of liquid across all training sessions in a day, a supplement of water was provided to ensure at least 1.0 ml total was received each day. In the early stages of training, head direction was partially automated by MouseoVeR to ensure that the mice could successfully navigate all regions of the mazes. As performance and skill on the spherical treadmill improved, we reduced the contribution of the automated heading component and correspondingly increased the manually controlled turning component. This increased the contribution of the subject’s running behavior to the control of head direction.

On days 3–5, mice continued to explore the same virtual maze over 2–3 training sessions per day, for a total of ~1 hr of in-maze time per day. On day 6, mice were exposed to a second, unique virtual maze, in addition to the initial maze, which was now familiar. For days 6–10, mice were trained to explore and become familiar with the second maze and the transitions between the two mazes. On day 11, after a minimum of 5 days of training in each of the virtual mazes, mice were considered ready for experimental recordings. From day 11 onwards, each mouse explored familiar and novel mazes during recording on 3–4 days, interleaved with additional training sessions or rest days.

### Electrophysiology

On recording days, trained mice were anesthetized with isoflurane (~1.5%,~0.8 l/min flow rate) and placed in a modified stereotaxic frame that clamps the head plate. If this was the first recording day from that hemisphere, a craniotomy (~1 mm^2^) was performed over dorsal CA1. Dura removal was performed with the assistance of collagenase (1 mg/ml in 1x PBS with 1.5 mM Ca, Collagenase type I, Sigma-Aldrich). Collagenase was applied for ~10–15 min, followed by application of bovine serum albumin solution (1 mg/ml in 1x PBS, Sigma Aldrich) for ~5 min, and finally rinsed with saline. A glass recording pipette (~2 MΩ) filled with saline was lowered into the brain and used to monitor the extracellular local field potential and unit activity in order to accurately map the depth of the dorsal CA1 pyramidal cell layer. Once the depth of the pyramidal layer was determined, the craniotomy was cleaned and rinsed with saline. To protect the brain during the recovery period, we created a sealed environment by filling a well surrounding the craniotomy with saline that was closed off with a cap lined with silicone (Kwik-Cast sealant, WPI, Sarasota, FL). All animals were mobile within a few minutes after being placed in their home cages. After a minimum of 1 hr of recovery time, mice were placed on the treadmill. Blind in vivo whole-cell recordings were obtained from the right or left dorsal CA1 pyramidal cell layer (Lee et al., 2009, 2014) using recording pipettes (5–7 MΩ) filled with an intracellular solution containing (in mM) K-gluconate 135, HEPES 10, Na_2_-phosphocreatine 10, KCl 4, MgATP 4, and Na_3_GTP 0.3 (pH adjusted to 7.2 with KOH) as well as biocytin (0.2%). After a gigaseal was obtained, the seal was held for up to ~4 min with no positive pressure to let the network recover before breaking into the membrane. After achieving the whole-cell configuration, the VR display was turned on and the mice were free to explore the mazes for sweetened water rewards as on training days. However, on recording days, in addition to the previously explored familiar mazes, mice could be exposed to a novel, previously unencountered, maze. Current-clamp measurements of V_m_ (amplifier low-pass filter set to 5 kHz) were sampled at 25 kHz. The animal’s behavior in the virtual maze was sampled at 30 Hz (the projector frame rate). All data analyzed is from recording periods with no holding current or no more than −20 pA of negative holding current applied to the pipette. Recordings were not corrected for the liquid junction potential. All neurons included in this study had the electrophysiological characteristics of somatic CA1 pyramidal whole-cell recordings (Lee et al., 2009; Epsztein et al., 2011; Lee et al., 2012, 2014). To minimize the number of animals used, we attempted to maximize the number of recordings per animal. Due to recording from multiple cells within a single animal across a window of ~5–10 days, we did not attempt to recover the histology of the recorded cells.

### Data analysis

All analysis was done using custom-written programs in Matlab. All novel versus familiar maze epoch comparisons as well as other comparisons (e.g., initial versus late lap amplitudes within epochs) were done using non-parametric paired (Wilcoxon signed-rank) and unpaired (Mann-Whitney U) tests assuming unequal variances, unless otherwise noted. Correspondingly, all numerical values in the text are reported as the median ± standard error of the median, unless otherwise noted, and boxplots show the 25–75%-iles and the median (horizontal black line). (Standard error of the median was computed as follows. From the sample population containing n samples, we drew n samples with replacement to create a sample group and then computed the median of the sample group. This was repeated 10,000 times to obtain 10,000 sample group medians. Finally, we computed the standard deviation of the sample group medians to obtain the standard error.) Pearson’s linear correlation coefficient was used to assess correlations between features, with significance determined with respect to the hypothesis that there was no correlation. All P-values reported are 2-sided. A value of p<0.05 was defined as statistically significant. Only temporally adjacent novel and familiar mazes were included in the analysis of NOV-FAM maze pairs. For example, if the order of presentation of mazes during the recording protocol was (1) FAM, (2) NOV, (3) FAM’, and (4) FAM2, then 2 NOV-FAM maze pairs were considered for analysis: FAM-NOV and NOV-FAM’. Analysis was limited to data collected during periods when the animal’s speed was ≥5 cm/s unless otherwise noted. The choice of dividing each epoch into early and late periods between laps 5 and 6 was based on previous extracellular studies showing that a substantial amount of the change in place field spiking activity during an epoch occurs within the first five laps (Mehta et al., 2000; Ekstrom et al., 2001).

Thirty-two cells were recorded from 15 mice navigating in virtual mazes. A maze was considered novel if it was the first day that the animal had encountered that maze (with the exception of 3 cases in which the animal had encountered the maze once on a previous day). For all familiar maze epochs, the animal had previously explored the maze a minimum of 19 times over the course of at least 5 days. Cells were active (where ‘active’ was defined as follows: peak AP firing rate as a function of location averaged over the maze epoch ≥0.5 Hz and spiking in ≥2 laps) in 47% of epochs in familiar mazes (21/45 epochs, 15/29 cells) and 69% of epochs in novel mazes (9/13 epochs, 8/12 cells). If active epochs were instead defined as those with an overall mean AP firing rate >0.1 Hz, the results were similar (NOV: 9/13, FAM: 18/45). The ~1.5 fold larger fraction of active epochs in novel environments agrees with the ~1.4 fold increased fraction of active dorsal CA1 pyramidal cells in rats exploring real-world novel versus familiar mazes (Karlsson and Frank, 2008).

### Subthreshold membrane potential and AP firing rate

The locations in the virtual mazes were linearized by collapsing them onto a long curve that went around the track, giving a 1-D representation of animal location. For display purposes, the beginning (position = 0 cm) and end (position =~135–240 cm) of the curve, which represent the same location, was chosen based on where the cellular activity was approximately the lowest across all time periods in the maze epoch (overall epoch average). Movement from zero to the end represents a full lap in the maze. The subthreshold V_m_ trace was estimated from the raw V_m_ trace as previously described (Epsztein et al., 2011; Lee et al., 2012). Briefly, all APs and any parts of the raw V_m_ trace directly attributable to the somatic spikes themselves (e.g., after-depolarizations) were removed, as well as the entirety of the slow, large, putatively calcium-based depolarization that often follows a burst of APs (which thus includes removing the entirety of each CS). The remaining trace was then linearly interpolated across the resulting gaps, yielding the subthreshold V_m_. The overall epoch mean AP firing rate and subthreshold V_m_ as a function of the animal’s virtual location were determined every 4 cm (‘spatial bin’) along the track, here using 12 cm-wide boxcar smoothing. The peak AP rate and peak subthreshold V_m_ were defined as the maximum spatially binned values. The baseline V_m_ (Figure 4B) was computed as the mean of the subthreshold V_m_ values for the 10% of spatial bins with lowest subthreshold V_m_. Similar operations were performed for individual laps to derive the AP rate, subthreshold V_m_, and peak and baseline V_m_ values for each lap. For each maze epoch, only directions in which the animal sampled each location on at least two different laps during the recording were considered for analysis. For bidirectional mazes, cellular activity in the direction that contained the largest overall epoch peak subthreshold V_m_ response was selected for comparisons between NOV-FAM maze pairs. For analysis of place fields or activity across laps within an epoch, each direction was included independently. Some laps were eliminated from analysis because of holding current outside of the −20 to 0 pA range, or because of within-lap continuous baseline shifts (e.g., possibly due to residual dialysis even after waiting ~4 min after seal formation before breaking in, or from sudden brain movements relative to the pipette, etc.). The original absolute lap numbers were used for analysis when intervening laps were eliminated.

### Determination of the place field region and amplitude of the subthreshold V_m_ hill

Place fields were determined for each epoch and direction as follows. From the epoch average firing rate map, the candidate place field was defined as the region containing the peak AP firing rate and contiguous bins with rate greater than or equal to the baseline rate plus 20% of the difference between the peak and baseline rate (where the baseline is the mean rate in the 10% of bins with lowest rate, which is generally 0 Hz). Then, candidate place fields with peak rate ≥0.5 Hz, at least 2 laps of spiking in-field, and an in-field/out-field average firing rate ratio ≥3 were classified as place fields. Unless noted as referring to the place field, the field was determined from the overall mean subthreshold V_m_ as a function of position in the epoch as follows. From this subthreshold function, the baseline V_m_ (Figure 4B) was determined as was the spatial bin with peak subthreshold V_m_. The set of contiguous position bins around this peak bin where the mean subthreshold V_m_ was greater than or equal to the baseline V_m_ plus 30% of the difference between the peak and baseline V_m_ was defined as the inside of the field. The amplitude of the subthreshold hill was computed by subtracting the baseline V_m_ from the peak value of the mean subthreshold V_m_. Similar operations were performed for each lap to derive within-lap baseline (Figure 4—figure supplement 1B) and peak (Figure 3—figure supplement 1A,right and 1C) V_m_ values.

### Determination of the spatial response amplitude of AP firing rate and subthreshold V_m_ for experience-dependent analysis

The in-field peak spatial activity in each lap within a maze epoch (Figure 3A and J) was determined as follows. For each maze epoch the peak AP rate and subthreshold V_m_ value in each lap were determined from the region surrounding (±3 spatial bins) the location of the overall epoch peak AP rate and subthreshold V_m_, respectively. Individual lap peak values from this in-field region were then averaged across a specific set of laps (e.g., laps 1–2 for the ‘initial laps’ in Figure 3A), or averaged across all laps to obtain a mean value for the maze epoch (Figure 3G–H). Individual lap peak values were also determined irrespective of location (‘lap peak’), then collapsed across laps to obtain a mean value for the maze epoch (Figure 3—figure supplement 1B–C). For peak subthreshold V_m_ activity in each lap within a maze epoch with respect to the place field (Figure 3B, E–F and I), we took the peak subthreshold V_m_ value in each lap inside the place field region determined from the overall epoch AP activity. Individual lap peak values from this place field region were then averaged across a specific set of laps (e.g., laps 1–2 for the ‘initial laps’ in Figure 3B), or averaged across all laps to obtain a mean value for the maze epoch (Figure 3I). In the case that one of the epochs in the maze pair (e.g. in Figure 3I) was active but did not have a place field or was silent, values for that epoch were computed with respect to the region surrounding (±3 spatial bins) the location of the overall epoch peak subthreshold V_m_.

To assess the effect of CSs on peak spatial activity within a maze epoch (Figure 6C), for all cells recorded in a FAM or a NOV maze, and for maze epochs that contained a CS, we first determined the location (lap number and spatial bin) of the first CS in the epoch. Then for each lap, we determined the peak AP rate and subthreshold V_m_ in the region surrounding (±3 spatial bins) the CS location. These lap peak activity values were then aligned to the lap (lap = 0) that contained the first CS. Similarly, for comparison with maze epochs that did not contain a CS, we determined the lap number and spatial bin containing the peak individual lap subthreshold V_m_ value. Then for each lap, we found the peak activity values within the region surrounding this subthreshold V_m_ value.

### Spatial correlation of the AP firing rate, subthreshold V_m_, and speed profile for experience-dependent analysis

For Figure 5, Figure 5—figure supplements 1A–C and and 2, for maze epochs in which there were at least 4 laps of activity, the stability of spatial tuning of the AP firing rate and, separately, subthreshold V_m_ was determined for each lap as follows. A lap correlation score was determined by computing the Pearson’s correlation coefficient between the activity across each location (i.e., each spatial bin in the maze) within the lap with the overall epoch activity. A mean maze correlation score was then determined by taking the mean of the individual lap scores. For bidirectional mazes, the direction with the largest amplitude overall epoch peak subthreshold V_m_ response was selected for comparison.

To assess the effect of CSs on spatial tuning within a maze epoch (Figure 6D), for all cells recorded in a FAM or a NOV maze, and for maze epochs that contained a CS, we first determined the lap number of the first CS in the epoch. For each lap, the spatial correlation scores were computed as described above and aligned to the lap (lap = 0) that contained the first CS. Similarly, for comparison with maze epochs that did not contain a CS, we determined the lap number containing the peak individual lap subthreshold V_m_ value and lap correlation scores were then aligned to that lap.

To assess the animal’s speed profile for each lap (Figure 5—figure supplement 1D), the spatial correlation was taken between each lap’s speed profile and the average speed profile for the overall epoch. For Figure 5—figure supplement 1E, for every pair of laps in a novel epoch, a spatial correlation between the subthreshold V_m_ of the 2 laps and between the speed profile of the 2 laps was computed. Then the correlation of these values across all pairs of laps in an epoch was computed. The distribution (i.e., median) of the correlation values (one per epoch) across all epochs was then compared to 0. In a related analysis, the relationship between the instantaneous speed of the animal and the instantaneous subthreshold V_m_ value was determined as follows. For each novel and familiar epoch, the correlation between instantaneous speed and corresponding subthreshold Vm was determined for all contiguous timestamps associated with speed >5 cm/s (which is the same speed threshold as is used in all the analysis). The distribution of correlation values (one per epoch) across all epochs was then compared to 0.

### Spectral analysis and standard deviation of the subthreshold V_m_

For Figure 3—figure supplement 2, each maze epoch (and each lap) was separated into periods when the animal was inside versus outside the field for that epoch. In the case of the theta (5–10 Hz) band, the subthreshold V_m_ trace was first reduced to a sampling rate of 500 Hz via decimation. Short-time Fourier transform (STFT) methods were applied to compute the average power in the 5–10 Hz band. Note that ~0.25 s of data were excluded before and after each of the crossing times between the inside and outside of the field to eliminate contamination due to the temporal extent of the STFT window. In the case of the gamma (25–100 Hz) band, a 25–160 Hz band-pass and 60 Hz notch filter were applied to the subthreshold V_m_ trace, the sampling rate was reduced to 2 kHz, and the STFT of the resulting trace was taken. Then, the periods 60 ms before to 60 ms after each AP and intracellular complex spike burst were removed, as well as 60 ms of data before and after each crossing time between the inside and outside of the field, and the power in the 25–100 Hz band was averaged over the remaining periods inside the field. For the standard deviation of the subthreshold V_m_ trace, a 1–160 Hz band-pass and a 60 Hz notch filter were applied, and periods 17 ms before to 17 ms after each AP and complex spike burst as well as crossing times were removed. Then, the standard deviation over the remaining periods inside the field was computed.

### Determination of spontaneous AP threshold

The spontaneous AP threshold (Figure 4A) for a given maze epoch was determined as previously described (Epsztein et al., 2011). Briefly, for each AP, we set the threshold to be the V_m_ value at which the dV/dt crossed 10 V/s (or 0.33 × the peak dV/dt of that AP, whichever was lower, in order to handle the slower APs that occurred later within bursts and CSs) on its way to the AP peak V_m_. Note that the threshold for individual APs varies with the level of depolarization before the AP (Figure 4—figure supplement 1A), and therefore we determined the threshold from a subset of APs that did not have APs immediately preceding them (i.e., isolated spikes and first spikes of bursts), that occurred during less depolarized periods, that did not possess a dV/dt shoulder (Epsztein et al., 2010), and that were not a part of a CS.

### Determination of input resistance

During maze exploration, we injected two 100-ms-long hyperpolarizing current steps of −0.2 nA separated by 500 ms every 20 s through the recording pipette (Figure 4C, left). To determine the mean input resistance (R_N_) within a maze epoch (Figure 4C), we first eliminated any V_m_ responses to the current steps that were masked by large spontaneous fluctuations, averaged the remaining responses during the epoch, and then applied a previously described procedure (Crochet and Petersen, 2006) to the average response. The specific steps used were as previously described (Epsztein et al., 2011).

### Analysis of evoked number of APs

During maze exploration, we injected one 100-ms-long depolarizing current step of +0.1–0.2 nA every 20 s through the recording pipette (Figure 4C, left). For each maze epoch, a single stimulus intensity was chosen to evoke APs, and the mean number of APs evoked per stimulus was normalized to the median value across all maze epochs in which the same stimulus intensity was used. For the comparison of evoked activity (Figure 4D), we only included data if the amplitude of the current step was the same for the FAM and NOV epochs of the maze pair.

### Shape of subthreshold V_m_ hill under place field

For Figure 5—figure supplement 4, the overall epoch AP rate and subthreshold V_m_ per lap as a function of position were determined every 2 cm along the track using 12 cm-wide boxcar smoothing for each epoch with a clear place field. From the AP rate function, the baseline was determined (mean of the AP rate values for the 10% of spatial bins with lowest AP rate, generally 0 Hz) as was the spatial bin with peak AP rate. The set of contiguous position bins around this peak bin where the mean AP rate was greater than or equal to the baseline plus 30% of the difference between the peak and baseline was defined as the inside of the AP rate field. The subthreshold V_m_ from each lap was taken for positions starting one-fourth of the width of the AP rate field before the start of the (AP rate) field to one-fourth of the field width after the end of the field, then averaged across the desired set of laps (e.g., laps 1–2 for the ‘initial’ period). Each such curve was interpolated at 60 evenly spaced position values, normalized in amplitude (0 = minimum subthreshold V_m_ value, 1 = maximum) and normalized with respect to the position along the field (0 to 1 for the inside of the field, −0.25 to 1.25 including the regions on each side of the field, and flipped if necessary so that the animal’s running direction was from −0.25 to 1.25), then averaged across the different epoch’s fields (mean ± SE).

### Complex spike occurrence

Classification of events as complex spikes (CSs) was done as previously described (Epsztein et al., 2011). Note that for the purposes of determining the location at which a CS occurred, we set the time of occurrence to be the time of the peak of the first AP in the CS. To assess the degree of association between novel place field formation and CSs, we considered all CSs whether or not they occurred when the animal was moving above or below the threshold speed (5 cm/s) and whether or not they occurred spontaneously or were triggered (i.e., evoked inadvertently by the 100-ms-long depolarizing current steps). In all other analyses involving CSs, we only included CSs that occurred spontaneously and when the animal was moving above the threshold speed.

## References

[bib1] Andersen P, Morris R, Amaral D, Bliss T, O’Keefe J (2007). The Hippocampus Book.

[bib2] Barry C, Ginzberg LL, O'Keefe J, Burgess N (2012). Grid cell firing patterns signal environmental novelty by expansion. PNAS.

[bib3] Basu J, Zaremba JD, Cheung SK, Hitti FL, Zemelman BV, Losonczy A, Siegelbaum SA (2016). Gating of hippocampal activity, plasticity, and memory by entorhinal cortex long-range inhibition. Science.

[bib4] Bennett C, Arroyo S, Hestrin S (2013). Subthreshold mechanisms underlying state-dependent modulation of visual responses. Neuron.

[bib5] Bi GQ, Poo MM (1998). Synaptic modifications in cultured hippocampal neurons: dependence on spike timing, synaptic strength, and postsynaptic cell type. Journal of Neuroscience.

[bib6] Bittner KC, Grienberger C, Vaidya SP, Milstein AD, Macklin JJ, Suh J, Tonegawa S, Magee JC (2015). Conjunctive input processing drives feature selectivity in hippocampal CA1 neurons. Nature Neuroscience.

[bib7] Blum KI, Abbott LF (1996). A model of spatial map formation in the Hippocampus of the rat. Neural Computation.

[bib8] Bostock E, Muller RU, Kubie JL (1991). Experience-dependent modifications of hippocampal place cell firing. Hippocampus.

[bib9] Cacucci F, Wills TJ, Lever C, Giese KP, O'Keefe J (2007). Experience-dependent increase in CA1 place cell spatial information, but not spatial reproducibility, is dependent on the autophosphorylation of the alpha-isoform of the calcium/calmodulin-dependent protein kinase II. Journal of Neuroscience.

[bib10] Cai DJ, Aharoni D, Shuman T, Shobe J, Biane J, Song W, Wei B, Veshkini M, La-Vu M, Lou J, Flores SE, Kim I, Sano Y, Zhou M, Baumgaertel K, Lavi A, Kamata M, Tuszynski M, Mayford M, Golshani P, Silva AJ (2016). A shared neural ensemble links distinct contextual memories encoded close in time. Nature.

[bib11] Chen G, King JA, Burgess N, O'Keefe J (2013). How vision and movement combine in the hippocampal place code. PNAS.

[bib12] Chiappe ME, Seelig JD, Reiser MB, Jayaraman V (2010). Walking modulates speed sensitivity in Drosophila motion vision. Current Biology.

[bib13] Crochet S, Petersen CC (2006). Correlating whisker behavior with membrane potential in barrel cortex of awake mice. Nature Neuroscience.

[bib14] D'Albis T, Jaramillo J, Sprekeler H, Kempter R (2015). Inheritance of hippocampal place Fields through Hebbian Learning: effects of Theta modulation and phase precession on structure formation. Neural Computation.

[bib15] Dudman JT, Tsay D, Siegelbaum SA (2007). A role for synaptic inputs at distal dendrites: instructive signals for hippocampal long-term plasticity. Neuron.

[bib16] Duncan K, Ketz N, Inati SJ, Davachi L (2012). Evidence for area CA1 as a match/mismatch detector: a high-resolution fMRI study of the human Hippocampus. Hippocampus.

[bib17] Dupret D, O'Neill J, Pleydell-Bouverie B, Csicsvari J (2010). The reorganization and reactivation of hippocampal maps predict spatial memory performance. Nature Neuroscience.

[bib18] Ekstrom AD, Meltzer J, McNaughton BL, Barnes CA (2001). NMDA receptor antagonism blocks experience-dependent expansion of hippocampal "place fields". Neuron.

[bib19] Epsztein J, Brecht M, Lee AK (2011). Intracellular determinants of hippocampal CA1 place and silent cell activity in a novel environment. Neuron.

[bib20] Epsztein J, Lee AK, Chorev E, Brecht M (2010). Impact of spikelets on hippocampal CA1 pyramidal cell activity during spatial exploration. Science.

[bib21] Frank LM, Stanley GB, Brown EN (2004). Hippocampal plasticity across multiple days of exposure to novel environments. Journal of Neuroscience.

[bib22] Fu Y, Tucciarone JM, Espinosa JS, Sheng N, Darcy DP, Nicoll RA, Huang ZJ, Stryker MP (2014). A cortical circuit for gain control by behavioral state. Cell.

[bib23] Giessel AJ, Sabatini BL (2010). M1 muscarinic receptors boost synaptic potentials and calcium influx in dendritic spines by inhibiting postsynaptic SK channels. Neuron.

[bib24] Golding NL, Staff NP, Spruston N (2002). Dendritic spikes as a mechanism for cooperative long-term potentiation. Nature.

[bib25] Grienberger C, Chen X, Konnerth A (2014). NMDA receptor-dependent multidendrite ca(2+) spikes required for hippocampal burst firing in vivo. Neuron.

[bib26] Guo ZV, Hires SA, Li N, O'Connor DH, Komiyama T, Ophir E, Huber D, Bonardi C, Morandell K, Gutnisky D, Peron S, Xu NL, Cox J, Svoboda K (2014). Procedures for behavioral experiments in head-fixed mice. PLoS One.

[bib27] Han JH, Kushner SA, Yiu AP, Cole CJ, Matynia A, Brown RA, Neve RL, Guzowski JF, Silva AJ, Josselyn SA (2007). Neuronal competition and selection during memory formation. Science.

[bib28] Harris KD, Hirase H, Leinekugel X, Henze DA, Buzsáki G (2001). Temporal interaction between single spikes and complex spike bursts in hippocampal pyramidal cells. Neuron.

[bib29] Harvey CD, Collman F, Dombeck DA, Tank DW (2009). Intracellular dynamics of hippocampal place cells during virtual navigation. Nature.

[bib30] Hasselmo ME (2006). The role of acetylcholine in learning and memory. Current Opinion in Neurobiology.

[bib31] Hebb DO (1949). The Organization of Behavior: A Neuropsychological Theory.

[bib32] Herkenham M (1978). The connections of the nucleus reuniens thalami: evidence for a direct thalamo-hippocampal pathway in the rat. The Journal of Comparative Neurology.

[bib33] Hill AJ (1978). First occurrence of hippocampal spatial firing in a new environment. Experimental Neurology.

[bib34] Hollup SA, Molden S, Donnett JG, Moser MB, Moser EI (2001). Accumulation of hippocampal place fields at the goal location in an annular watermaze task. Journal of Neuroscience.

[bib35] Hölscher C, Schnee A, Dahmen H, Setia L, Mallot HA (2005). Rats are able to navigate in virtual environments. Journal of Experimental Biology.

[bib36] Johnston D, Christie BR, Frick A, Gray R, Hoffman DA, Schexnayder LK, Watanabe S, Yuan LL (2003). Active dendrites, potassium channels and synaptic plasticity. Philosophical Transactions of the Royal Society B: Biological Sciences.

[bib37] Johnston D, Narayanan R (2008). Active dendrites: colorful wings of the mysterious butterflies. Trends in Neurosciences.

[bib38] Kamondi A, Acsády L, Wang XJ, Buzsáki G (1998). Theta oscillations in Somata and dendrites of hippocampal pyramidal cells in vivo: activity-dependent phase-precession of action potentials. Hippocampus.

[bib39] Kandel ER, Spencer WA (1961). Electrophysiology of hippocampal neurons. II. After-potentials and repetitive firing. Journal of Neurophysiology.

[bib40] Karlsson MP, Frank LM (2008). Network dynamics underlying the formation of sparse, informative representations in the Hippocampus. Journal of Neuroscience.

[bib41] Kemp A, Manahan-Vaughan D (2007). Hippocampal long-term depression: master or minion in declarative memory processes?. Trends in Neurosciences.

[bib42] Kentros C, Hargreaves E, Hawkins RD, Kandel ER, Shapiro M, Muller RV (1998). Abolition of long-term stability of new hippocampal place cell maps by NMDA receptor blockade. Science.

[bib43] Kentros CG, Agnihotri NT, Streater S, Hawkins RD, Kandel ER (2004). Increased attention to spatial context increases both place field stability and spatial memory. Neuron.

[bib44] Larkin MC, Lykken C, Tye LD, Wickelgren JG, Frank LM (2014). Hippocampal output area CA1 broadcasts a generalized novelty signal during an object-place recognition task. Hippocampus.

[bib45] Lee AK, Epsztein J, Brecht M (2009). Head-anchored whole-cell recordings in freely moving rats. Nature Protocols.

[bib46] Lee D, Lin BJ, Lee AK (2012). Hippocampal place fields emerge upon single-cell manipulation of excitability during behavior. Science.

[bib47] Lee D, Shtengel G, Osborne JE, Lee AK (2014). Anesthetized- and awake-patched whole-cell recordings in freely moving rats using UV-cured collar-based electrode stabilization. Nature Protocols.

[bib48] Leutgeb S, Leutgeb JK, Barnes CA, Moser EI, McNaughton BL, Moser MB (2005). Independent codes for spatial and episodic memory in hippocampal neuronal ensembles. Science.

[bib49] Leutgeb S, Leutgeb JK, Treves A, Moser MB, Moser EI (2004). Distinct ensemble codes in hippocampal areas CA3 and CA1. Science.

[bib50] Lever C, Wills T, Cacucci F, Burgess N, O'Keefe J (2002). Long-term plasticity in hippocampal place-cell representation of environmental geometry. Nature.

[bib51] Li S, Cullen WK, Anwyl R, Rowan MJ (2003). Dopamine-dependent facilitation of LTP induction in hippocampal CA1 by exposure to spatial novelty. Nature Neuroscience.

[bib52] Lim S, McKee JL, Woloszyn L, Amit Y, Freedman DJ, Sheinberg DL, Brunel N (2015). Inferring learning rules from distributions of firing rates in cortical neurons. Nature Neuroscience.

[bib53] Lisman JE, Grace AA (2005). The hippocampal-VTA loop: controlling the entry of information into long-term memory. Neuron.

[bib54] Lisman JE (1997). Bursts as a unit of neural information: making unreliable synapses reliable. Trends in Neurosciences.

[bib55] Liu X, Ramirez S, Pang PT, Puryear CB, Govindarajan A, Deisseroth K, Tonegawa S (2012). Optogenetic stimulation of a hippocampal engram activates fear memory recall. Nature.

[bib56] Lovett-Barron M, Turi GF, Kaifosh P, Lee PH, Bolze F, Sun XH, Nicoud JF, Zemelman BV, Sternson SM, Losonczy A (2012). Regulation of neuronal input transformations by tunable dendritic inhibition. Nature Neuroscience.

[bib57] Maimon G, Straw AD, Dickinson MH (2010). Active flight increases the gain of visual motion processing in Drosophila. Nature Neuroscience.

[bib58] Mankin EA, Sparks FT, Slayyeh B, Sutherland RJ, Leutgeb S, Leutgeb JK (2012). Neuronal code for extended time in the Hippocampus. PNAS.

[bib59] Markram H, Lübke J, Frotscher M, Sakmann B (1997). Regulation of synaptic efficacy by coincidence of postsynaptic APs and EPSPs. Science.

[bib60] McHugh TJ, Blum KI, Tsien JZ, Tonegawa S, Wilson MA (1996). Impaired hippocampal representation of space in CA1-specific NMDAR1 knockout mice. Cell.

[bib61] McNamara CG, Tejero-Cantero Á, Trouche S, Campo-Urriza N, Dupret D (2014). Dopaminergic neurons promote hippocampal reactivation and spatial memory persistence. Nature Neuroscience.

[bib62] McNaughton BL, Barnes CA, O'Keefe J (1983). The contributions of position, direction, and velocity to single unit activity in the Hippocampus of freely-moving rats. Experimental Brain Research.

[bib63] Mehta MR, Barnes CA, McNaughton BL (1997). Experience-dependent, asymmetric expansion of hippocampal place fields. PNAS.

[bib64] Mehta MR, Lee AK, Wilson MA (2002). Role of experience and oscillations in transforming a rate code into a temporal code. Nature.

[bib65] Mehta MR, Quirk MC, Wilson MA (2000). Experience-dependent asymmetric shape of hippocampal receptive fields. Neuron.

[bib66] Milstein AD, Bloss EB, Apostolides PF, Vaidya SP, Dilly GA, Zemelman BV, Magee JC (2015). Inhibitory gating of Input comparison in the CA1 microcircuit. Neuron.

[bib67] Monaco JD, Rao G, Roth ED, Knierim JJ (2014). Attentive scanning behavior drives one-trial potentiation of hippocampal place fields. Nature Neuroscience.

[bib68] Morris RG, Garrud P, Rawlins JN, O'Keefe J (1982). Place navigation impaired in rats with hippocampal lesions. Nature.

[bib69] Morris RG (1989). Synaptic plasticity and learning: selective impairment of learning rats and blockade of long-term potentiation in vivo by the N-methyl-D-aspartate receptor antagonist AP5. Journal of Neuroscience.

[bib70] Muller RU, Kubie JL (1987). The effects of changes in the environment on the spatial firing of hippocampal complex-spike cells. Journal of Neuroscience.

[bib71] Niell CM, Stryker MP (2010). Modulation of visual responses by behavioral state in mouse visual cortex. Neuron.

[bib72] Nitz D, McNaughton B (2004). Differential modulation of CA1 and dentate gyrus interneurons during exploration of novel environments. Journal of Neurophysiology.

[bib73] O'Keefe J, Conway DH (1978). Hippocampal place units in the freely moving rat: why they fire where they fire. Experimental Brain Research.

[bib74] O'Keefe J, Dostrovsky J (1971). The Hippocampus as a spatial map. preliminary evidence from unit activity in the freely-moving rat. Brain Research.

[bib75] Penley SC, Hinman JR, Long LL, Markus EJ, Escabí MA, Chrobak JJ (2013). Novel space alters theta and gamma synchrony across the longitudinal axis of the hippocampus. Frontiers in Systems Neuroscience.

[bib76] Polack PO, Friedman J, Golshani P (2013). Cellular mechanisms of brain state-dependent gain modulation in visual cortex. Nature Neuroscience.

[bib77] Rajasethupathy P, Sankaran S, Marshel JH, Kim CK, Ferenczi E, Lee SY, Berndt A, Ramakrishnan C, Jaffe A, Lo M, Liston C, Deisseroth K (2015). Projections from neocortex mediate top-down control of memory retrieval. Nature.

[bib78] Ranck JB (1973). Studies on single neurons in dorsal hippocampal formation and septum in unrestrained rats. I. behavioral correlates and firing repertoires. Experimental Neurology.

[bib79] Rashid AJ, Yan C, Mercaldo V, Hsiang HL, Park S, Cole CJ, De Cristofaro A, Yu J, Ramakrishnan C, Lee SY, Deisseroth K, Frankland PW, Josselyn SA (2016). Competition between engrams influences fear memory formation and recall. Science.

[bib80] Ravassard P, Kees A, Willers B, Ho D, Aharoni D, Cushman J, Aghajan ZM, Mehta MR (2013). Multisensory control of hippocampal spatiotemporal selectivity. Science.

[bib81] Rich PD, Liaw HP, Lee AK (2014). Place cells. large environments reveal the statistical structure governing hippocampal representations. Science.

[bib82] Royer S, Zemelman BV, Losonczy A, Kim J, Chance F, Magee JC, Buzsáki G (2012). Control of timing, rate and bursts of hippocampal place cells by dendritic and somatic inhibition. Nature Neuroscience.

[bib83] Rubin A, Geva N, Sheintuch L, Ziv Y (2015). Hippocampal ensemble dynamics timestamp events in long-term memory. eLife.

[bib84] Savelli F, Knierim JJ (2010). Hebbian analysis of the transformation of medial entorhinal grid-cell inputs to hippocampal place fields. Journal of Neurophysiology.

[bib85] Schneider DM, Nelson A, Mooney R (2014). A synaptic and circuit basis for corollary discharge in the auditory cortex. Nature.

[bib86] Scoville WB, Milner B (1957). Loss of recent memory after bilateral hippocampal lesions. Journal of Neurology, Neurosurgery and Psychiatry.

[bib87] Seelig JD, Chiappe ME, Lott GK, Dutta A, Osborne JE, Reiser MB, Jayaraman V (2010). Two-photon calcium imaging from head-fixed Drosophila during optomotor walking behavior. Nature Methods.

[bib88] Sheffield ME, Dombeck DA (2015). Calcium transient prevalence across the dendritic Arbour predicts place field properties. Nature.

[bib89] Sheth A, Berretta S, Lange N, Eichenbaum H (2008). The amygdala modulates neuronal activation in the Hippocampus in response to spatial novelty. Hippocampus.

[bib90] Takahashi H, Magee JC (2009). Pathway interactions and synaptic plasticity in the dendritic tuft regions of CA1 pyramidal neurons. Neuron.

[bib91] Takeuchi T, Duszkiewicz AJ, Sonneborn A, Spooner PA, Yamasaki M, Watanabe M, Smith CC, Fernández G, Deisseroth K, Greene RW, Morris RG (2016). Locus coeruleus and dopaminergic consolidation of everyday memory. Nature.

[bib92] Teles-Grilo Ruivo LM, Mellor JR (2013). Cholinergic modulation of hippocampal network function. Frontiers in Synaptic Neuroscience.

[bib93] Thompson LT, Best PJ (1990). Long-term stability of the place-field activity of single units recorded from the dorsal Hippocampus of freely behaving rats. Brain Research.

[bib94] Traub RD, Llinás R (1979). Hippocampal pyramidal cells: significance of dendritic ionic conductances for neuronal function and epileptogenesis. Journal of Neurophysiology.

[bib95] Tsien JZ, Huerta PT, Tonegawa S (1996). The essential role of hippocampal CA1 NMDA receptor-dependent synaptic plasticity in spatial memory. Cell.

[bib96] Vertes RP (2015). Major diencephalic inputs to the Hippocampus: supramammillary nucleus and nucleus reuniens. Circuitry and function. Progress in Brain Research.

[bib97] Wilson MA, McNaughton BL (1993). Dynamics of the hippocampal ensemble code for space. Science.

[bib98] Wong RK, Prince DA (1978). Participation of calcium spikes during intrinsic burst firing in hippocampal neurons. Brain Research.

[bib99] Xu L, Anwyl R, Rowan MJ (1998). Spatial exploration induces a persistent reversal of long-term potentiation in rat Hippocampus. Nature.

[bib100] Xu W, Morishita W, Buckmaster PS, Pang ZP, Malenka RC, Südhof TC (2012). Distinct neuronal coding schemes in memory revealed by selective erasure of fast synchronous synaptic transmission. Neuron.

[bib101] Zhou Y, Won J, Karlsson MG, Zhou M, Rogerson T, Balaji J, Neve R, Poirazi P, Silva AJ (2009). CREB regulates excitability and the allocation of memory to subsets of neurons in the amygdala. Nature Neuroscience.

[bib102] Ziv Y, Burns LD, Cocker ED, Hamel EO, Ghosh KK, Kitch LJ, El Gamal A, Schnitzer MJ (2013). Long-term dynamics of CA1 hippocampal place codes. Nature Neuroscience.

